# A bulk acoustic resonator with vertical electrodes for wideband filters

**DOI:** 10.1038/s44172-026-00733-1

**Published:** 2026-07-21

**Authors:** Silvan Stettler, Edgar Navarro-Gessé, Carlos Collado, Jordi Mateu, Luis Guillermo Villanueva

**Affiliations:** 1 Advanced Nanoelectromechanical Systems Laboratory, École Polytechnique Fédérale de Lausanne (EPFL), Lausanne, Switzerland; 2 Department of Signal Theory and Communications, Universitat Politècnica de Catalunya (UPC), Barcelona, Spain

**Keywords:** Electrical and electronic engineering, Mechanical engineering, NEMS

## Abstract

Radiofrequency front ends for current and next generation (5G and 6G) wireless communication demand acoustic filters that combine wide bandwidth, high power capability, and thermal stability. Existing surface and bulk acoustic wave technologies face inherent trade-offs between electromechanical coupling, lithographic tunability, and robustness. Here, we introduce the bulk acoustic resonator with vertical electrodes (VBAR), a device that combines the advantages of suspended and solidly mounted resonators. VBARs use lithium niobate ridges with sidewall electrodes to excite a shear-horizontal bulk acoustic resonance, providing frequency control through lithography in a configuration that is mechanically anchored to the substrate. Fabricated VBARs exhibit electromechanical coupling coefficients exceeding 30% in the 2–4 GHz range, enabling ladder filters with fractional bandwidths of nearly 20%. While further optimization is necessary to minimize losses, the VBAR concept offers an alternative route toward wideband and robust radiofrequency filters for next-generation wireless systems.

## Abstract (web summary)

Silvan Stettler and colleagues introduce a bulk acoustic resonator technology based on lithium niobate for radiofrequency filtering applications. These devices exhibit electromechanical coupling coefficients over 30%, enabling filters with fractional bandwidths near 20% for next-generation wireless communications.

## Introduction

Currently, the radiofrequency (RF) filter market is dominated by surface acoustic wave (SAW) and bulk acoustic wave (BAW) technologies, which have been continually improved to meet evolving mobile communication standards. The roll-out of the fifth-generation (5G) standard has created a renewed demand for microacoustic filters capable of delivering higher frequencies, enhanced fractional bandwidths (FBW), low temperature coefficients of frequency (TCF), and increased power handling^1^. Some bands in the sub-6GHz spectrum (5G Frequency Range 1, 5G-FR1) demand not only higher frequency than previous LTE bands, but also much larger fractional bandwidths (FBW, up to 24% for n77). In addition, GPP3 Release 17 introduced power class 1.5, which specifies increased output powers of 29 dBm for some of those bands (i.e., n77, n78)^2^. These specifications have been driving the progress of SAW and BAW resonator technology in recent years, each offering distinct advantages in certain aspects while exhibiting limitations in others. For common filter topologies, such as the ladder filter, resonance frequency (*f*_r_), electromechanical coupling coefficient (*k*_t_^2^), and quality factor (*Q*) of the individual resonator building block relate to the center frequency (*f*_c_), fractional bandwidth (FBW), and insertion loss (IL) of the filter^3^.

For SAW resonators, *f*_r_ can be scaled lithographically by varying the pitch of the interdigital transducer (IDT) electrodes. This dependence makes SAW technology extremely versatile, as it enables the design of multiple filtering components with different *f*_c_ on the same die (e.g., for multiplexers) by changing the IDT layout. In addition, the most widely used SAW substrates are strong piezoelectrics, such as lithium niobate (LiNbO_3_) or lithium tantalate (LiTaO_3_), which can provide large *k*_t_^2^ (>20%), resulting in FBWs of over 15% in a ladder filter^4^. Unfortunately, the use of conventional SAW technology based on bulk piezoelectric substrates for frequencies > 2.5  GHz (necessary for 4G and 5G) is complicated by losses from bulk-wave radiation and low acoustic phase velocities (*v*_p_). In this regard, one of the key innovations in recent years has been the introduction of thin film piezoelectric on insulator (POI) substrates^5–7^. These substrates consist of a thin film of monocrystalline LiNbO_3_/LiTaO_3_ bonded to a high velocity substrate, such as high-resistivity Si^8–10^, quartz^11,12^, sapphire^13^, or silicon carbide (SiC)^14–18^ and have led to significant improvements of the quality factor (*Q*), *k*_t_^2^, and resonance frequencies > 2.5 GHz. Even though the lithographic resolution is still a limiting factor, POI substrates have enabled SAW-based filters with low IL above 5 GHz^19^.

In contrast to SAW, the frequency of BAW resonators, such as free-standing bulk acoustic resonators (FBAR) or solidly mounted BAW resonators (BAW-SMR), is not lithography-dependent but is controlled by the thickness of the piezoelectric film. Commercial BAW products rely on AlN and scandium-doped AlN (AlScN), which can be deposited in sub-micron thickness layers to reach frequencies up to 8 GHz and above^20–22^. The intrinsic piezoelectric coupling factor of AlScN limits the *k*_t_^2^ of such BAW resonators to around 20%, thereby restricting the wide-bandwidth potential. It has been shown that LiNbO_3_-based BAW resonators can achieve much higher *k*_t_^2^ (>30%) than all previously mentioned technologies. However, integrating monocrystalline LiNbO_3_ films into an FBAR or BAW-SMR structure requiring metal layers below the film poses technological challenges^23–26^. Laterally excited bulk acoustic resonators (XBARs) have emerged as a promising solution in that regard^27–31^. XBARs benefit from the high *k*_t_^2^ of the shear BAW in LiNbO_3_ and scalability to high frequency, but do not require a metal layer below the suspended piezoelectric film^29,32,33^. Despite its advantages, the XBAR architecture, which employs a suspended LiNbO_3_ film, is not conducive to sustaining high power levels^34^. In comparison, solidly mounted technologies, such as BAW-SMR and POI-based SAW, are inherently more robust, and heat can be dissipated away from the surface owing to the high thermal conductivity of commonly used substrates (Si ~ 140 W/(K ∙ m), SiC > 400 W/(K ∙ m)). In a suspended film, heat generated under high power can only be dissipated laterally through the film (or through the narrow electrodes), and to exacerbate the issue, LiNbO_3_ is a poor thermal conductor ( < 10 W/(K ∙ m)).

In this paper, we explore an alternative resonator structure^35^ that aims to combine high electromechanical coupling (*k*_t_^2^ > 30%) with a solidly mounted configuration and lithographic frequency control. Recently, it was shown that vertical Si fins attached to the substrate and with piezoelectric transducers on the sidewalls can support lithographically defined BAW resonances^36^, albeit with low *k*_t_^2^. In other recent work, a LiNbO_3_ fin was covered with IDT electrodes to excite a fin-confined acoustic resonance, demonstrating a *k*_t_^2^ of 6.2% at 300 MHz^37^. Here, we introduce the bulk acoustic resonator with vertical electrodes (VBAR), which consists of LiNbO_3_ piezoelectric ridges with metal electrodes deposited on the sidewalls. The resonance frequency is defined lithographically by the ridge width, and we experimentally demonstrate that (lateral shear) BAW resonances with *k*_*t*_^*2*^ exceeding 30% in the 2–4 GHz range can be excited in such a structure. Furthermore, we show that ladder filters built from VBARs can achieve FBWs close to 20%. Furthermore, we investigate the TCF and behavior of the VBARs under high power (including non-linearity). Despite remaining technological challenges, we show that VBARs can sustain input power levels on the order of 30 dBm.

## Results

### Vertical electrode bulk acoustic resonator (VBAR) concept

**Fig. 1:**
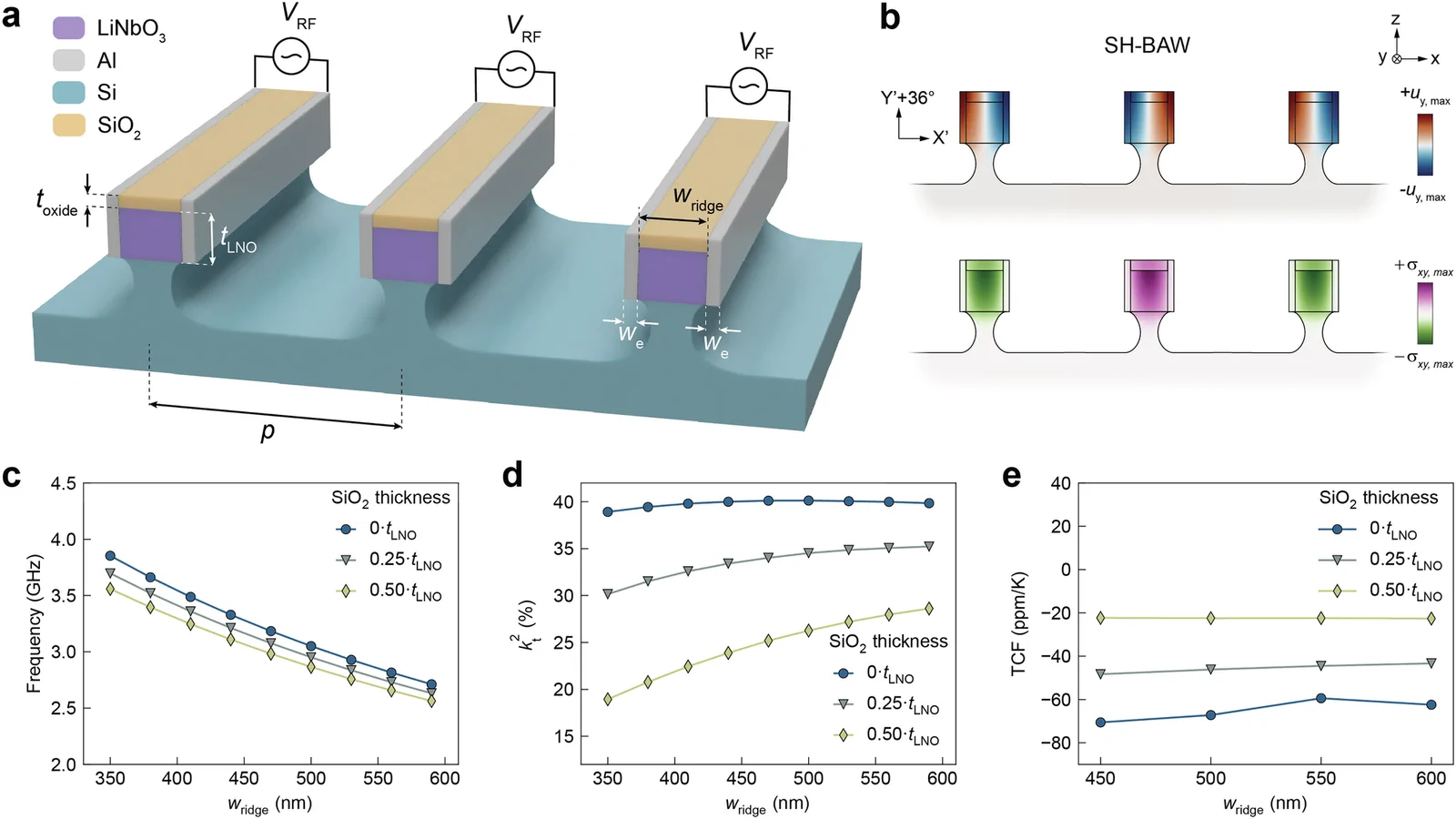
Vertical electrode bulk acoustic resonator (VBAR) working principle. **a** Schematic of a VBAR composed of multiple parallel-connected ridges of YX36°-cut LiNbO_3_ and SiO_2_ with vertical Al electrodes on the ridge sidewalls. **b** Mode shape of the shear horizontal bulk acoustic wave (SH-BAW) resonance. *u*_y_ is the out-of-plane displacement (along y) and σ_xy_ is the shear horizontal component of the stress tensor. X’ and Y’ denote the crystalline axes of LiNbO_3_. **c–e** Simulated **c** resonance frequency, **d** electromechanical coupling (*k*_t_^2^), and **e** first-order temperature coefficient of the resonance frequency of the SH-BAW mode for varying relative thicknesses of the SiO_2_ layer (*t*_oxide_). The electrode width (*w*_e_) is fixed to 100 nm and the LiNbO_3_ thickness (*t*_LNO_) is 400 nm.

Figure 1a depicts a schematic illustration of the resonator architecture we propose. The resonating elements consist of an array of piezoelectric ridges that are attached to a silicon substrate via a narrow Si pedestal. A layer of aluminum deposited on the vertical sidewalls of the ridges forms the electrodes to drive the resonator. To address the temperature stability of the resonator, a layer of SiO_2_ can optionally be integrated on top of the ridge.

Instead of confining the acoustic wave between the horizontal planar surfaces of a suspended membrane (as in FBARs, XBARs), the resonating mode in VBARs is a standing wave confined by the vertical sidewalls of the ridge. Such a configuration differs from BAW resonators utilizing thickness-defined BAWs. The resonance frequency of VBARs is set by the lithographically controllable width of the ridges (*w*_ridge_) rather than by the film thickness. In addition to the planar footprint (i.e., the number of parallel-connected ridges), the static capacitance (*C*_0_) of a VBAR can be scaled by increasing the thicknesses of the LiNbO_3_ and SiO_2_ layers. In contrast to resonators employing suspended films, anchoring the ridges to the substrate provides a path for dissipating heat generated by losses and offers increased mechanical stability, which may improve power-handling capacity.

Since the driving electric field is applied in-plane, we use YX36°-cut LiNbO_3_ as a piezoelectric material, which possesses strong electromechanical coupling coefficients for shear acoustic waves for excitation with in-plane electric fields (*e*_16_)^38^. In Fig. 1b, we show the mode shape of the main resonant mode, which is a shear-horizontal bulk acoustic wave (SH-BAW). The SH-BAW is characterized by displacement that is out-of-plane (*u*_y_, along y in Fig. 1b) and a stress field with a primarily shear horizontal component (σ_xy_). The half wavelength (λ/2) of the SH-BAW roughly corresponds to the total width of the acoustic cavity consisting of the LiNbO_3_ ridge and the electrodes. The width of the LiNbO_3_ ridge represents the critical lithographic dimension. The geometry of the Si pedestal, serving as an anchor for the ridges, plays a key role in achieving optimal acoustic performance. Solidly mounted resonators, such as BAW-SMR or POI SAW typically use a Bragg reflector stack^39,40^ or operate below the cut-off frequency of the BAWs in the substrate^41^ to confine the acoustic energy in the piezoelectric layer. Instead, we reduce acoustic energy leakage to the substrate by creating a constriction in the anchor beneath the LiNbO_3_ ridge. In this unique case of shear vibrations, where the resonant mode's displacement is primarily parallel to the resonator boundaries, a constriction can inhibit BAW transmission to the substrate. We note that this approach does not require substrates with high acoustic velocities, such as SiC or diamond and can be implemented with cheaper Si substrates. The mode shape in Fig. 1b shows that the displacement at resonance is well confined in the acoustic cavity. In the Supplementary Information, Section 1, we further discuss the rationale of this structure and illustrate the influence of the anchor geometry on *Q*, *k*_t_^2^, and *f*_r_. Indeed, in addition to minimizing acoustic losses, a narrow anchor allows the LiNbO_3_ domains to deform freely, thereby maximizing *k*_t_^2^. Since the narrow anchors isolate the resonating elements from the substrate, the individual VBAR ridges in the array are acoustically independent of each other. Therefore, the pitch (*p*) of the array does not influence the resonance frequency and can be chosen arbitrarily large, which may facilitate the etching and electrode deposition processes required to fabricate the structure.

In Fig. 1c, we show that the resonance frequency can be tuned by adjusting *w*_ridge_. Assuming Al electrodes with a width of 100 nm on either side, the simulations predict that *f*_r_ can be varied from 2.5 to 4 GHz for values of *w*_ridge_ between 350 and 600 nm. If a layer of SiO_2_ is added on top of the LiNbO_3_ for temperature compensation, the phase velocity of the acoustic cavity is lowered, thereby shifting the resonance to slightly lower frequencies. Figure 1d shows the simulated *k*_t_^2^ for the same range of *w*_ridge_ and *t*_oxide_. Due to the strong *e*_16_ piezoelectric coefficient in YX36°-cut LiNbO_3_, our simulations predict *k*_t_^2^ close to 40% without a SiO_2_ layer. If the latter is included, we observe a *k*_t_^2^ decrease that is approximately proportional to the relative thickness of SiO_2_. On the other hand, in Fig. 1e we show that the addition of SiO_2_ can significantly improve the TCF of the SH-BAW mode. Al, Si (at low doping concentrations), and especially LiNbO_3_ have relatively large negative temperature coefficients of stiffness, which results in a TCF of roughly −70 ppm/K or worse for the uncompensated SH-BAW mode. With its positive temperature coefficient, the SiO_2_ compensates for the negative temperature coefficient of the other materials involved, and we simulate that the TCF can be improved to around −30 ppm/K.

Since the main SH-BAW mode propagates across the width of the ridge, the absolute thickness of the LiNbO_3_ (*t*_LNO_) is not the primary factor that determines *f*_r_ and *k*_t_^2^. The choice of *t*_LNO_ has a significant impact on *C*_0_ and the appearance of thickness-confined spurious modes. The electrode width (*w*_e_) contributes to the total width of the acoustic cavity and thus affects the achievable *f*_r_ and *k*_t_^2^ for a given ridge width. Further discussion and simulations that elucidate the impact of the layer thicknesses and electrode width are given in the Supplementary Information, Section 2.

### VBAR implementation and characterization

The experimental implementation of VBARs poses unique challenges not commonly encountered in more standard BAW or SAW resonator architectures. First, to accurately control the resonant frequency, the width of the ridges forming the resonating body must be uniform across the structure's thickness. As with SAW devices, this requires a well-controlled lithography process, but also a method to pattern the constituent layers with vertical sidewalls. Second, to minimize acoustic energy leakage to the substrate, a narrow anchor beneath the ridge is required. Removing Si from underneath a device is common in fabrication processes for acoustic resonators or MEMS in general and can be achieved with several methods such as XeF_2_ gas, SF_6_ plasma or potassium hydroxide. For VBARs, the undercut needs to be controlled with sub-micron precision to attain less than half of *w*_ridge_ to avoid excessive substrate leakage, but not much less, otherwise the ridges may be released entirely. Moreover, the undercut must be the same for all individual ridges that make up a VBAR to ensure they resonate at the same frequency. This process is difficult due to the high etch rate and non-uniformity of many of the release processes (i.e., XeF_2_). We developed a fabrication process flow for VBARs that addresses those challenges, and we give a detailed description in the Methods.

**Fig. 2:**
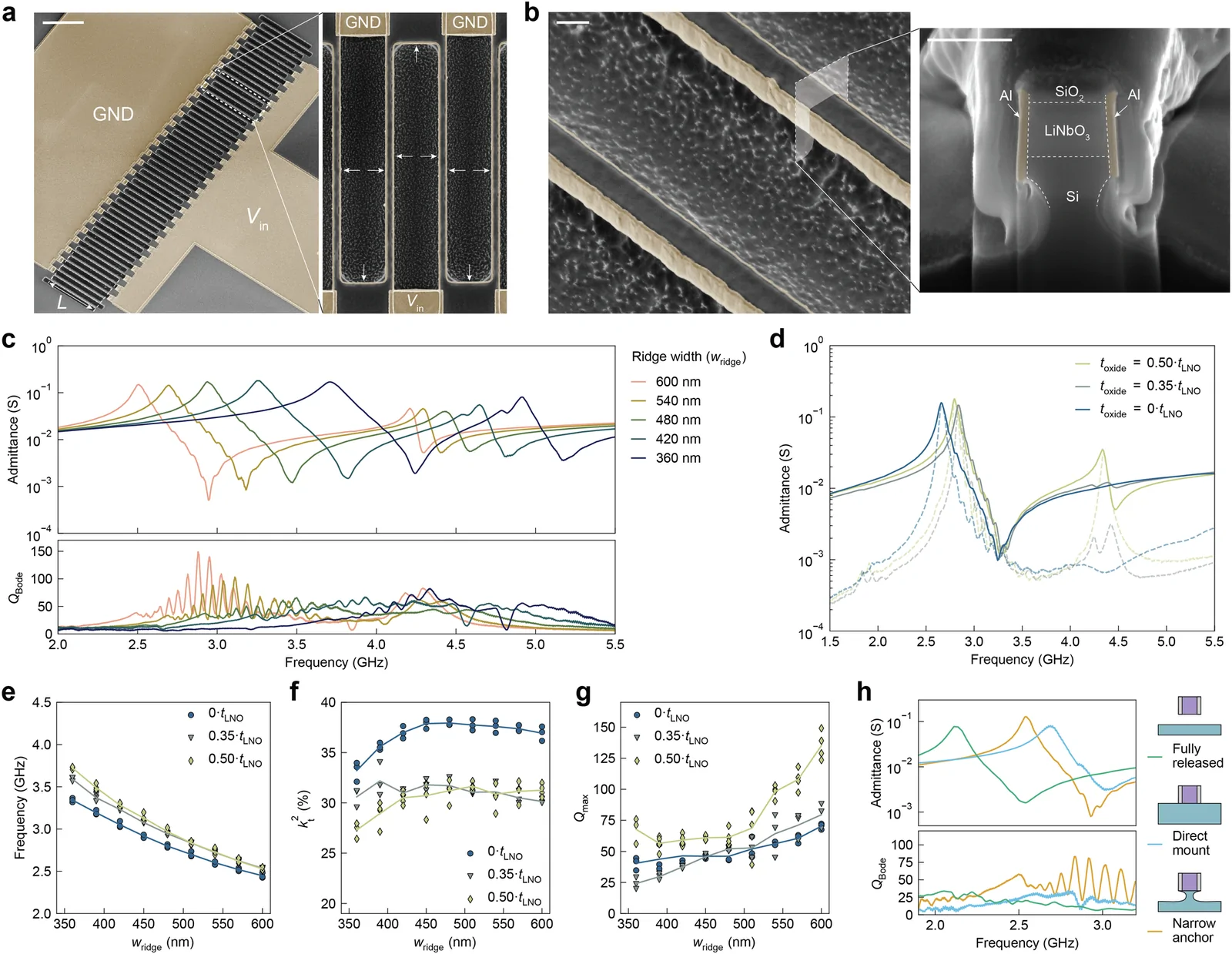
VBAR implementation and characterization. **a** False-color SEM micrographs of a fabricated one-port VBAR. Aluminum features are artificially shaded in dark yellow. The annotations indicate the polarity of the applied RF signal and aperture length (*L*). The tips of the arrows indicate the thin electrodes on the ridge sidewalls. Scale bar 10 µm. **b** False-color SEM micrograph showing close-up and cross-sectional views of the resonating ridges. Scale bar 500 nm. **c** Measured admittance and *Q*_Bode_ for VBARs with varying *w*_ridge_ (*t*_oxide_ = 0.5∙*t*_LNO_ = 200 nm). **d** Measured admittance of three VBARs with different SiO_2_ thicknesses, showing the effect of that layer on the magnitude of a spurious mode relatively close to anti-resonance. **e–g** Extracted **e** resonance frequency, **f** *k*_t_^2^, and **g** maximum *Q*_Bode_ (*Q*_max_) of the measured VBARs with *L* = 70∙*w*_ridge_ and varying SiO_2_ thicknesses. Markers represent all individually measured raw values to show the full data distribution. Solid lines indicate the mean values for *n* = 3 identical resonators (*n* = 4 for *t*_oxide_ = 0.50∙*t*_LNO_). **h** Measured admittance of a VBAR (*w*_ridge_ = 600 nm) with a narrow anchor (as shown in **b**) compared to fully released and directly mounted devices.

Figure 2a shows SEM micrographs of a fabricated one-port VBAR. We define the resonating ridges by patterning an array of large openings separated by the desired *w*_ridge_ into the substrate. The etching process used for the ridges results in vertical sidewalls (85° to 90°, see Fig. 2b and Fig. S7 in the Supplementary Information). Next, we deposit a layer of Al conformally on the patterned substrate. With another lithography step, we cover the regions that form the pads and subsequently remove the Al from all exposed planar surfaces by ion-beam etching. Following this self-aligned process, a thin film of Al remains on the sidewalls of the previously patterned holes, forming a ring that is connected on one edge to the pads. Hence, the structures between the holes form an array of parallel-connected VBAR ridges. With the connection pattern shown in Fig. 2a, adjacent ridges are always driven with opposite phases (see also mode shapes in Fig. 1b). The fabrication is finalized by etching the partial undercut of the VBAR ridges. In Fig. 2b, we show a close-up view of two ridges of the fabricated VBAR, and a cross-sectional view obtained after cutting a ridge with Focused Ion Beam (FIB) milling. The cross-sectional view shows that the Si anchor is substantially narrower than the LiNbO_3_ ridge. The undercut is the same for all VBARs on the same substrate and is determined by the etching duration. This means that the Si anchor is wider or narrower relative to the ridge width for lower- or higher-frequency devices. The Al electrodes have a width of approximately 70 nm and widen slightly in the lower half of the ridge. In Fig. 2c, we show the measured admittance (*Y*_11_) of VBARs with varying ridge widths, yielding resonance frequencies in the 2.5 to 4 GHz range with distances between *f*_r_ and the anti-resonance frequency (*f*_ar_) on the order of 500 MHz. We observe that the measured *Q*s (that is, Bode *Q*, *Q*_Bode_, see also in the Methods) are generally below 150 and become gradually worse for narrower ridges and higher frequencies. Since the relative width of the anchors is largest for the lowest *f*_r_, this implies that leakage to the substrate is not a dominating factor in the observed *Q*s. Above *f*_ar,_ a prominent spurious peak arises from a higher-order SH-BAW mode with thickness dependence (see Supplementary Information, Section 2). This mode can be suppressed by adjusting the SiO_2_ thickness (see Fig. 2d). Between *f*_r_ and *f*_ar,_ we observe evidence of transversal modes in the form of closely spaced ripples (see Fig. S4 in the Supplementary Information). In Fig. 2e–g, we summarize the extracted *f*_r_, *k*_t_^2^, and *Q*_max_. Qualitatively, the observed *f*_r_ and *k*_t_^2^ match with the idealized simulations shown in Fig. 1. Non-uniformities in the thickness of the layers involved and other deviations from the initially conceived geometry (such as the sidewall angle and vertical position of electrodes) factor into the apparent discrepancies. In contrast to the simulations, we observe a decrease in *f*_r_ when removing the SiO_2_ layer. Since Al is not etched during the SiO_2_ removal process, the electrodes extend above the LiNbO_3_ ridge, which lowers the frequency compared to the simulated case (flush electrode and ridge top surface, see Fig. S8 in the Supplementary Information). With *k*_t_^2^ of 30% or more, VBARs could potentially be useful for wide-bandwidth filters in the 5G mid-range. Even so, the quality factors we measure (Fig. 2g) are a major roadblock for a filter with low insertion loss. VBARs with the widest ridges (and as such, the lowest *f*_r_) and largest total thickness exhibit the highest *Q*s. This trend suggests that the high losses could originate from surface loss mechanisms, such as surface roughness, which are key factors determining *Q* in thin film LiNbO_3_ resonators^42^ and nanoelectromechanical resonators in general^43^. This is supported qualitatively by the roughness of the sidewall that is visible in the SEM micrographs (Fig. 2b), as well as the high surface-to-volume ratio inherent to the nearly square cross-section of the ridges. However, we currently lack a quantitative measurement (i.e., AFM) of the roughness of the sidewalls. Thus, attributing the limited *Q* primarily to surface roughness remains a hypothesis for future studies. At *f*_r_, the *Q* is further reduced by the resistive losses in the electrodes, which account for roughly half of the impedance at resonance. To experimentally study the effect of different anchor widths on *Q* (see the simulated trend in Supplementary Fig. S2), we are limited by the etching process, which yields a constant undercut for all resonators on a given chip and is impractical to vary device to device. Instead, we demonstrate the energy confinement mechanism by fabricating the extreme cases where the ridges are either directly mounted to the substrate or fully released (Fig. 2h). In the directly mounted configuration, the peak admittance is significantly lower compared to the standard VBAR with an anchor. As suggested by our simulations, the acoustic energy is poorly confined for the directly mounted configuration, and therefore, the measured *Q*_Bode_ never exceeds 30. Even though fully releasing the ridge eliminates the possibility of substrate leakage, it also further increases the surface area. We observe a lower *Q*_Bode_ for the fully released resonators compared to the standard configuration with an anchor. This supports the notion that using a narrow anchor configuration, substrate leakage is largely suppressed and not the predominant reason for the overall low *Q*s.

**Fig. 3:**
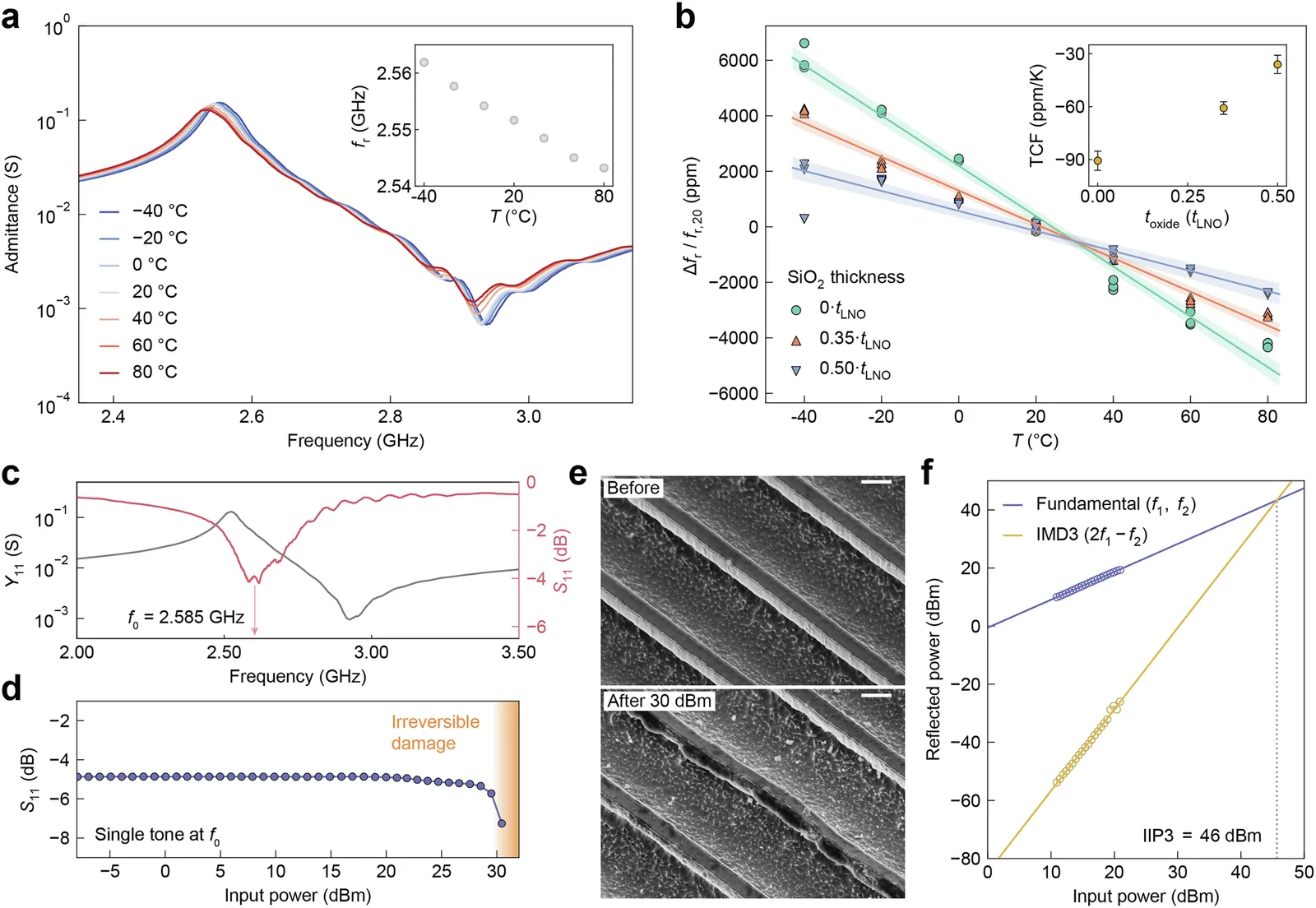
Measured temperature coefficient of frequency (TCF) and behavior at high power. **a** Admittance response and extracted *f*_r_ for varying temperature (*T*). **b** Extracted relative shift of *f*_r_ with respect to *f*_r_ at 20 °C for VBARs with *w*_ridge_ = 600 nm and varying SiO_2_ thicknesses. For every *T*, we measure each resonator 3 times to minimize the influence of the contact on the TCF extraction. We perform linear fits to extract the first-order TCF from the slope of the fitted line. The solid lines represent the mean fitted values, and the error bands indicate the 95% confidence intervals for the mean regression lines. Inset: Extracted mean slope values representing the TCF (markers) and their 95% confidence intervals (error bars). **c–f** Power handling and non-linearity measurements of a VBAR with *w*_ridge_ = 600 nm, *t*_oxide_ = 0.35∙*t*_LNO_, *L* = 70∙*w*_ridge_ and *N* = 50 ridges. **c** Admittance (*Y*_11_) and reflection coefficient (*S*_11_) measured at low power (−15 dBm) before applying a high-power tone at *f*_0_. **d** *S*_11_ (of the resonator and probe) at *f*_0_ for increasing input power. Starting at 29.5 dBm, we observe irreversible damage to the resonator. **e** SEM micrographs of a VBAR before and after subjecting the device to a 30 dBm tone. Scale bar 1 µm**. f** One-port measurement of the IMD3 in the reflected signal with two input tones at *f*_1_ = 2.617 GHz and *f*_2_ = *f*_1_ + 2 MHz. The linear extrapolations of the reflected fundamental and IMD3 power indicate a IIP3 value of 46 dBm.

In Fig. 3a, we show the admittance of a VBAR measured at temperatures ranging from -40 to 80°C. The measurements are performed in a vacuum, but we do not see any notable increase in *Q* compared to atmospheric pressure. In Fig. 3b, we show the relative change in resonance frequency with respect to room temperature for VBARs with different thicknesses of SiO_2_. The inclusion of SiO_2_ lowers the TCF from −90 to −36 ppm/K, which corresponds in relative terms to the improvement predicted in simulation (−68 to −27 ppm/K). Next, we assess the power handling capabilities of the VBARs. In Fig. 3c, we show the reflection coefficient and corresponding admittance of a VBAR measured at low power (−15 dBm) before applying high-power signals. The return loss (RL) is at least 4 dB for a narrow band just above resonance, which indicates that the resonator under test absorbs a significant portion of the incoming power at those frequencies due to the rather low *Q*s. Using an in-house setup (described in Section 5 of the Supplementary Information), we apply a high-power tone at *f*_0_ where the RL is maximized and measure the reflected power. Figure 3d shows the measured *S*_11_ of the resonator and probe as a function of incident power. Up to 29.5 dBm, *S*_11_ is within 1 dB of the value measured at low power. Above 30 dBm, the resonators are irreversibly damaged and are eventually destroyed completely. We inspect the damaged resonator after the high-power test (Fig. 3e). For incident power levels approaching 30 dBm (1 W, corresponding approximately to 0.6 W dissipated in the resonator based on the *S*_11_ at *f*_0_, see Fig. 3c), the Al electrodes show signs of melting, and the ridges visibly deform, which may be due to the Si anchor breaking. In addition, using a two-tone excitation in a one-port reflection configuration, we measure the third-order intermodulation distortion (IMD3) in the reflected signal arising from nonlinearities of the resonator (Fig. 3f). With the two tones centered at 2.618 GHz, we obtain an IIP3 value of 46 dBm. Further details on the power handling and IMD3 measurements are available in the Supplementary Information, Section 5.

### VBAR filters

**Fig. 4:**
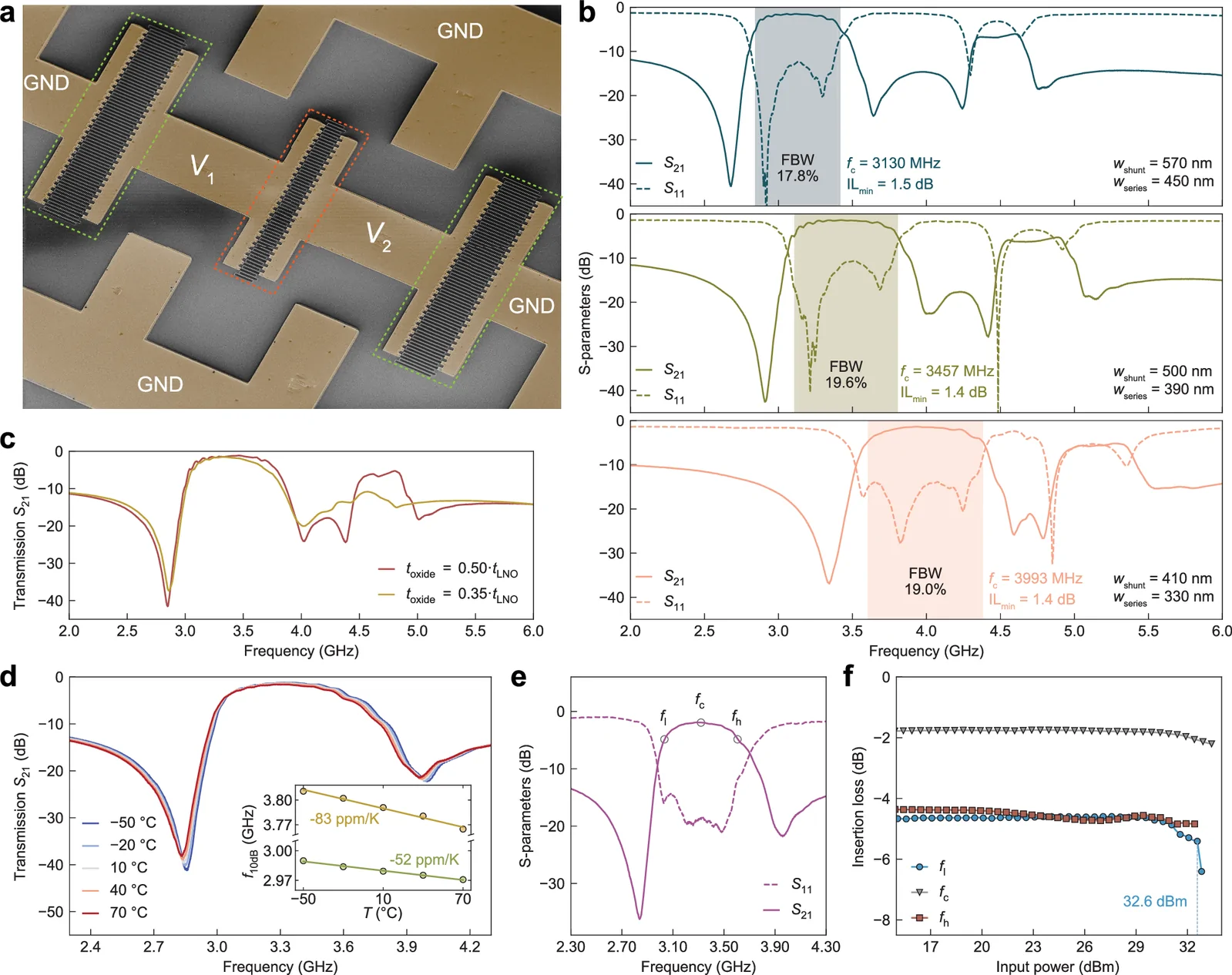
Bandpass filters using VBAR building blocks. **a** False-color SEM micrographs of a fabricated filter consisting of one series (red frame) and two shunt (green frame) VBARs. **b** Measured filter responses for three filters with different center frequencies (*f*_c_). *f*_c_ is set by lithographically varying *w*_ridge_ of the series and shunt resonators (inset text). The aperture length (*L*) and number of ridges (*N*) of the resonators are adjusted to achieve a *C*_0_ of approximately 810 fF (shunt) and 460 fF (series). The resulting dimensions are *L* = 70∙*w*_ridge_, *N* = 60 (shunt) and *L* = 40∙*w*_ridge_, *N* = 50 (series). For these three filters, *t*_oxide_ = 0.5∙*t*_LNO_. **c** S_21_ of identical filters with different *t*_oxide_. **d** Change in *S*_21_ with temperature and extracted TCF of the 10 dB passband edges for a VBAR filter with *t*_oxide_ = 0.35∙*t*_LNO_. **e**,**f** Power handling tests of a VBAR filter. **e** Filter response measured at low power (−15 dBm) before applying high-power tones in the center of the passband (*f*_c_) and at the band edges (*f*_l_, *f*_h_). **f** Insertion loss at these three frequencies for increasing input power. The filter is destroyed when the input power at the lower band edge (*f*_l_) exceeds 32.6 dBm.

We implement bandpass filters with VBAR impedance elements to demonstrate their potential for wide-bandwidth, multi-frequency filtering applications. As shown in Fig. 4a, we adopt a π-type filter topology containing two identical shunt resonators and one series resonator. We lithographically adjust *w*_ridge_ of the VBARs to align *f*_ar_ of the shunt to *f*_r_ of the series resonator and control the center frequency of the filters. Crucially, we do not require any trimming of the film thicknesses to achieve the required frequency shifts. In Fig. 4b, we show the measured reflection and transmission coefficients (*S*_11_ and *S*_21_) for three filters with center frequencies ranging from 3.1 to 4 GHz, which we fabricate on the same chip. Due to the high *k*_t_^2^ of the VBARs, the FBWs of the main passband due to the SH-BAW resonances can reach nearly 20%. The spurious passband on the right of the intended passband is associated with the higher-order SH-BAW mode discussed previously (see Fig. 2d). Using a thinner SiO_2_ layer reduces its excitation and improves the upper-stopband rejection (Fig. 4c). The filters shown in Fig. 4b have minimum insertion losses of only 1 to 2 dB despite the relatively high overall losses in the resonators. However, this is mainly due to the choice of topology with a single series resonator, which in turn provides little out-of-band rejection. Improvements in *Q* could drastically improve the flatness and roll-off slope of the passband and enable low insertion loss also in a higher-order filter. To complement the TCF and high-power characterization on single resonators (Fig. 3), we characterize the transmission response of the filter for varying temperatures (Fig. 4d) and extract the TCF at the 10 dB edges of the passband. We find that the TCF of the lower edge is comparable to the resonance TCF measured for a single resonator with similar SiO_2_ thickness. Furthermore, we assess the power handling capabilities of a VBAR filter by measuring the insertion loss for high-power tones located in the center and at the edges of the passband (Fig. 4e, f). Compared to the previous tests (Fig. 3c–e) where all the input power is directed at a single resonator, the filter topology and frequency of the applied tone heavily influence how much power is dissipated in the individual resonators in the filter^44^. We observe that the IL of the filter gradually starts to change at around 30 dBm of input power, indicating the onset of non-linear behavior. Ultimately, the filter fails completely when the input power applied at the lower passband edge exceeds 32.6 dBm.

## Discussion

We have reported on an acoustic resonator architecture that attempts to combine the respective advantages of suspended and solidly mounted resonator configurations. We have shown that VBARs can deliver levels of *k*_t_^2^ (>30%) that are comparable with fully suspended shear acoustic wave resonators, such as XBARs or LiNbO_3_ FBARs, while arguably providing better mechanical robustness due to the solidly mounted configuration of the resonator. Enabled by the ability to achieve large relative shifts in *f*_r_ through lithographic design control, we have shown that VBARs are suitable for implementing filters with FBWs close to 20% without increasing fabrication complexity.

Further, we have shown that VBARs can operate at power levels close to 30 dBm at the single-resonator level. While this is still considerably less than what commercial SAW^45^ and AlScN-based BAW^21,44,46^ resonators can achieve, it is a promising first result given that the losses in the demonstrated prototypes are relatively high. In general, the acoustic losses in the resonator are the most significant roadblock that must be overcome before VBARs can be considered as fully suitable for more complex filter topologies. Given the *Q*s we have demonstrated, it would be challenging to design filters that meet all the requirements for mid-range 5G bands. It is probable that the power handling could also be improved if the losses and thus heat generation in the resonators are minimized.

Several intrinsic challenges of the vertical geometry must be addressed to further develop the technology. The large surface-to-volume ratio of the present thin-film implementation increases the sensitivity to surface roughness or other fabrication imperfections. From a design perspective, using thicker LiNbO_3_ films on the order of a micron or more may be beneficial for VBARs. The lower surface-to-volume ratio may weaken the potential influence of a lossy surface, and the sensitivity of the resonance frequency to non-uniformities in the anchor dimensions would also be reduced. In any case, further optimization of the fabrication process to reduce the roughness of the interfaces and electrode sidewalls is desirable. An example of such an improvement could be replacing sputtering of the electrode metal with atomic layer deposition (ALD). The implementation of VBARs with substantially thicker LiNbO_3_ introduces the additional challenge of deeper LiNbO_3_ etching. Addressing this will require further process development to establish an etching method that not only achieves the required depth and mask selectivity but also maintains smooth, vertical sidewalls.

Provided these challenges can be successfully addressed, the concept of a non-planar resonator offers a combination of functionalities that cannot be readily achieved with conventional planar structures. We have shown that such a structure supports BAW resonances with higher *k*_t_^2^ than SAW resonances in LiNbO_3_, while also providing lithographic frequency control similar to SAW devices, which enables relatively straightforward implementation of filter prototypes. Furthermore, the general idea of a vertical, non-planar BAW resonator implies that the devices' static capacitance could be decorrelated from the resonator's planar footprint. Unlike FBARs or XBARs, the capacitance of such a resonator could be scaled by varying the thickness of the constitutive layers without significantly affecting the resonance frequency. This provides an additional degree of flexibility, enabling denser integration by reducing filter size or distributing heat over a larger substrate area under high power.

## Methods

### Thin film LiNbO_3_ on silicon substrates

The substrates used for fabrication consist of a thin film of LiNbO_3_ with a nominal thickness of 400 nm bonded to a high-resistivity silicon wafer purchased from NGK Insulators.

### Fabrication process flow

Fig. S5 in the Supplementary Information schematically outlines the process flow. As a first step, we deposit 1.7 microns of SiO_2_ using plasma-enhanced chemical vapor deposition (PECVD, Corial D250L), which serves as a hard mask layer for the LiNbO_3_ etching process later on. We pattern the deposited SiO_2_ layer using e-beam lithography, followed by an inductively coupled plasma (ICP) based dry etching using a C_4_F_8_/H_2_/He chemistry (SPTS Advanced Plasma System). Since the linewidth of the patterned SiO_2_ features subsequently translates into the width of the LiNbO_3_ features (i.e., the ridge width *w*_ridge_), we use e-beam lithography for this mask to ensure high uniformity. We split the subsequent SiO_2_ etching process into two successive steps with adjusted wafer bias and H_2_ flow to achieve sufficient selectivity to enable 1.7 µm etch depth into SiO_2_ with a 600 nm thick ZEP 520 A e-beam resist mask (see Fig. S7 in the Supplementary Information). We etch the exposed LiNbO_3_ using ion beam etching (IBE, Veeco Nexus IBE350) with a fixture angle of 45°, which results in vertical sidewalls for the LiNbO_3_ ridges (see Fig. S8 in the Supplementary Information). Next, we deposit and pattern a 300 nm Al layer serving as pads or filter interconnects with optical lithography and lift-off. To form the electrodes on the sidewalls, we first conformally deposit a 70 nm thick Al layer with sputtering. With another lithography step, we cover the pads and bus lines with photoresist. Subsequently, we use IBE with a fixture angle resulting in nearly vertical ion incidence (10°) to remove the previously sputtered Al in the exposed areas. This step removes Al only on horizontal surfaces (i.e., on top and next to the previously patterned LiNbO_3_ ridges). The Al film on the vertical sidewalls of the ridges is not etched during this process because the ion incidence angle is nearly parallel to the sidewalls. The next processing step is to create the Si pedestal that anchors the LiNbO_3_ ridges to the substrate. We achieve this with an ICP-based dry etching step with SF_6_ and a low-frequency wafer-voltage bias (Alcatel AMS 200 SE, see Fig. S7 in the Supplementary Information). For samples without a SiO_2_ layer, the final processing step is the removal of the remaining SiO_2_ covering the LiNbO_3_ ridges with a selective dry etch with a C_4_F_8_/H_2_/He chemistry (Fig. S9 in the Supplementary Information).

### RF measurements

All low-power RF measurements are carried out with a Rohde & Schwarz ZNB20 Vector Network Analyzer (VNA) with a power of −15 dBm. For measurements at room temperature, MPI Titan T26 GSG probes with 200 microns pitch are used. For the variable temperature measurements, we use a LakeShore CRX-4K cryogenic probe station with cryo-compatible Picoprobe GSG probes (GGB Industries). Before all measurements, we calibrate the setup using a calibration substrate and a standard open-short-match (OSM) scheme. From the measured data, we compute the performance parameters of the resonator with *k*_t_^2^ = π/2·*f*_r_/*f*_ar_·cot(π/2·*f*_r_/*f*_ar_) and *Q*_Bode_ = ω ∙ |*dS*_11_/*d*ω | ·1/(1- | *S*_11_ | ^2^)^47^. The setups for the high-power and non-linearity measurements are described in detail in the Supplementary Information, Section 5. We note that the non-linear behavior (IMD3, IIP3) of the filters was not characterized, as their passband frequencies exceed the 2.7 GHz upper limit of our non-linearity measurement setup.

## Supplementary information

Transparent Peer Review file

Supplementary Information: A bulk acoustic resonator with vertical electrodes for wideband filters

## Data Availability

The data shown in the plots have been deposited in the Zenodo database^48^.
