# Supplementary material for: A bulk acoustic resonator with vertical electrodes for wideband filters
Source: Commun Eng. 2026 Jul 21;5:193. doi: 10.1038/s44172-026-00733-1 (PMC13754800; doi:10.1038/s44172-026-00733-1)
Supplement: Supplementary file 1 [file 44172_2026_733_MOESM1_ESM.pdf]

nature portfolio

Peer Review File

A bulk acoustic resonator with vertical electrodes for wideband filters

Corresponding Author: Dr Silvan Stettler

This file contains all reviewer reports in order by version, followed by all author rebuttals in order by version.

Version 0:

Reviewer comments:

Reviewer #1

(Remarks to the Author)
This article presents an interesting implementation of a bulk acoustic resonator with vertical electrodes (VBAR), using lithium niobate ($LiNbO_3$) ridges with sidewall electrodes to excite a shear-horizontal bulk acoustic resonance. The fabricated VBARs exhibit electromechanical coupling coefficients exceeding 30% in the 2–4 GHz range, enabling ladder filters with fractional bandwidths of nearly 20%. This marks a new attempt following SAW, BAW, and XBAR technologies, holding significant implications for 5G communication filters. It might be of some archival value. The reviewer believes more effort is needed before it can meet the standards for publication.
1.The author mentions in the manuscript that YX36°-cut LN is used. It would be helpful to label the crystal orientation (XYZ) in the schematic of Figure 1(a) or (b) for clarity.
2.Electrodes are applied to the sidewalls of the $LiNbO_3$ ridges to excite in-plane propagating SH waves, as shown in Fig. S1. From the perspective of the crystal material, which piezoelectric coefficient is primarily utilized here? For example, in Z-cut LN, the e_{15} coefficient is typically used to excite the A_1 mode.
3.The author mentions in the text that in VBAR technology, "the pitch of the array does not influence the resonance frequency and can be chosen arbitrarily large to facilitate fabrication." While this simplifies lithography for the pitch, the critical dimension for frequency control—the Wridge—remains extremely challenging, requiring lithography at ~600 nm, which poses significant fabrication demands. Currently, surface acoustic wave (SAW) technology based on LN/SiC can achieve frequencies around ~7 GHz while also offering a high keff2. In light of this, what does the author consider to be the key advantages of the VBAR approach when evaluated comprehensively?
4.Given the relatively low Q observed in the fabricated resonators, which may be attributed to factors such as sidewall roughness and cross-sectional tilt angle of the ridges, has the sidewall roughness been characterized? Alternatively, have any comparative experiments with different etching angles been conducted?
5.How is the width of Al electrodes controlled, and does this parameter significantly affect device performance?
6.In Figure 2(c), the trace corresponding to a Wridge of 600 nm appears relatively faint and somewhat indistinct. If convenient, it would be helpful to slightly darken its color for better visual clarity.
7.The author mentions in the text that "the static capacitance of a VBAR can be scaled by increasing the thicknesses of the $LiNbO_3$ and SiO_2 layers." What are the respective static capacitance values of the series and shunt resonators in the filter, and what are the corresponding device parameters? The paper does not provide a clear mapping between the static capacitance and the relevant device parameters.

Reviewer #2

(Remarks to the Author)
The paper reports an impressive demonstration of novel GHz resonators in thin film lithium niobate. The most remarkable part is the inclusion of a filter demonstration. The thorough analysis is also impressive. The work will have a significant impact; therefore, it should be published after minor revisions.

- One major point is how the device will perform when it is fully suspended. The authors have compared solidly mounted and partially released. The main remaining uncertainty is how the Q, TCF, and IMD3 will change upon release. However, the reviewer recognizes that the authors may not wish to do so, given that the paper is still under review and that they may want to repurpose the device for other explorations. The reviewer suggests that the authors consider doing so at the end of the review process or expanding this discussion for a future conference publication. The paper should still be publishable,

provided that a brief comment on this is included in the manuscript.

- The IMD3 measurement should be better performed for the filter, as it is less measurable or comparable for resonators, due to 1) the high reflection nature and 2) the dramatic impedance contrast of the resonator. The IMD3 filter is a better measure of the matrix. The two-port IMD3 measurement also aligns more closely with the definition. The authors should consider reporting that instead. However, as noted in the previous comment, the paper is publishable without this; it would be strengthened.

- Could the authors comment on why the SH0 mode, but not the S0/A1/S1 mode are used here? The reviwer assumes that A1/S1 might not be confined, but that S0 might be at some risk? That would make the frequency even higher.

- The side wall might be the bottleneck here. Have the authors considered alternative metalization methods? The reviewer recalls ALD used for those HZO finbar devices. Would that be applicable here?

- Good to comment on how to develop further the technology, e.g., the intrinsic challenges of the structure and potential ways to overcome those.

- The fabrication process is quite impressive. Could the authors include more images of the devices at the intermediate fabrication steps in the supplementary document? The reviewer is interested in seeing the entire process and assumes readers will be as well.

Reviewer #3

(Remarks to the Author)
It is an interesting work. I have several questions and comments:
1. For power handling measurement (Fig. 3d), what is the actual power going through the resonator? What is the power density? It is better to add the SEM images of the damaged resonator after the power test.
2. The frequency uniformity of each pair is good. How to achieve it? Is this due to good fabrication, or is there coupling between each pair in one resonator?
3. How about TCF and the power handling of the filter?
4. Do you have the data to support the effect of the size of the Si anchor on the quality factor?
5. What is the limitation in improving the selectivity of the filter?

Version 1:

Reviewer comments:

Reviewer #1

(Remarks to the Author)
I thank the authors for their detailed rebuttal and improvements to the manuscript. My previous comments on crystallographic orientation, piezoelectric coefficients, and capacitance mapping have been adequately addressed, clarifying the VBAR's operating principles. I recommend publication after a few minor revisions to further contextualize the device's current limitations.

1. The hypothesis that surface roughness limits the quality factor is reasonable but lacks quantitative verification, such as via AFM. Please add a brief statement in the manuscript explicitly acknowledging this limitation and clarifying that attributing low Q to surface losses remains a hypothesis for future study.

2. Although experimental data for varying electrode widths is currently absent , it is a fundamental parameter that affects performance. Please add a brief theoretical discussion or simulated trend explaining how electrode thickness variations are expected to influence on f and Kt

Reviewer #2

(Remarks to the Author)
The paper is well edited and should be published. The paper is a good innovation in the area.

Reviewer #3

(Remarks to the Author)
The authors have answered most of my questions in the response letter, but they did not include the responses in their manuscript to improve clarity.
Please allow me to repeat the question:
1. For power handling measurement (Fig. 3d), what is the actual power going through the resonator? What is the power density? It is better to add the SEM images of the damaged resonator after the power test.
need to add the corresponding information to the manuscript

2. The frequency uniformity of each pair is good. How to achieve it? Is this due to good fabrication, or is there coupling between each pair in one resonator?
need to add the corresponding information to the manuscript
3. How about TCF and the power handling of the filter?
need to mention in the manuscript why these data were not measured
4. Do you have the data to support the effect of the size of the Si anchor on the quality factor?
need to mention in the manuscript why the measured data were challenging
5. What is the limitation in improving the selectivity of the filter?
need to show the simulation results to demonstrate the possibility of improving the selectivity

Version 2:

Reviewer comments:

Reviewer #1

(Remarks to the Author)
The authors have thoroughly addressed the concerns raised in the previous two rounds。 The newly added content in the revised manuscript, like the simulated trends for electrode width, the newly measured TCF and high-power data for the filter, all significantly improve the paper. The VBAR concept is novel and promising for wideband 5G/6G filters, and I recommend acceptance for publication.

Reviewer #3

(Remarks to the Author)
The authors have addressed all questions. I think the current version can be published

Open Access This Peer Review File is licensed under a Creative Commons Attribution 4.0 International License, which permits use, sharing, adaptation, distribution and reproduction in any medium or format, as long as you give appropriate credit to the original author(s) and the source, provide a link to the Creative Commons license, and indicate if changes were made.
In cases where reviewers are anonymous, credit should be given to 'Anonymous Referee' and the source.
The images or other third party material in this Peer Review File are included in the article’s Creative Commons license, unless indicated otherwise in a credit line to the material. If material is not included in the article’s Creative Commons license and your intended use is not permitted by statutory regulation or exceeds the permitted use, you will need to obtain permission directly from the copyright holder.
To view a copy of this license, visit https://creativecommons.org/licenses/by/4.0/

Advanced NEMS Group
EPFL-STI-IGM NEMS
MED 2 2726, Station 9
CH-1015, Lausanne Switzerland

Prof. Guillermo Villanueva
Guillermo.Villanueva@epfl.ch
Ph.: +41 21 693 11 87
http://nems.epfl.ch

Manuscript Number: COMMSENG-25-0857

Title: A bulk acoustic resonator with vertical electrodes for wideband filters

Authors: S. Stettler, E. Navarro-Gesse, C. Collado, J. Mateu, and L. G. Villanueva

February 14th, 2026

Dear Editor and Referees,

Thank you very much for reviewing our manuscript. Below, we have tried to address the comments that you made, and we changed the manuscript accordingly. We think that the changes made have contributed to the overall quality of the manuscript so we would like to thank the reviewers for their insightful comments and suggestions.

Legend: Reviewer's comments are typed in black over a gray background. Our responses are typed in black without any background shading. Changes are highlighted with a red font color. The line numbers refer to the clean revised version of the manuscript (without changes marked up).

Reviewer #1

This article presents an interesting implementation of a bulk acoustic resonator with vertical electrodes (VBAR), using lithium niobate ($LiNbO_3$) ridges with sidewall electrodes to excite a shear-horizontal bulk acoustic resonance. The fabricated VBARs exhibit electromechanical coupling coefficients exceeding 30% in the 2–4 GHz range, enabling ladder filters with fractional bandwidths of nearly 20%. This marks a new attempt following SAW, BAW, and XBAR technologies, holding significant implications for 5G communication filters. It might be of some archival value. The reviewer believes more effort is needed before it can meet the standards for publication.

Comment 1:

The author mentions in the manuscript that YX36°-cut LN is used. It would be helpful to label the crystal orientation (XYZ) in the schematic of Figure 1(a) or (b) for clarity.

Response:

We are grateful to the reviewer for this suggestion. We added a small minimal sketch to Fig. 1b and updated the legend. The most important point with regards to the crystalline orientation is that the electric field is applied along the crystalline X axis (denoted as X' in Fig. 1b) for maximum k_t^2.

Changes:

- Added crystalline axes to Fig. 1b

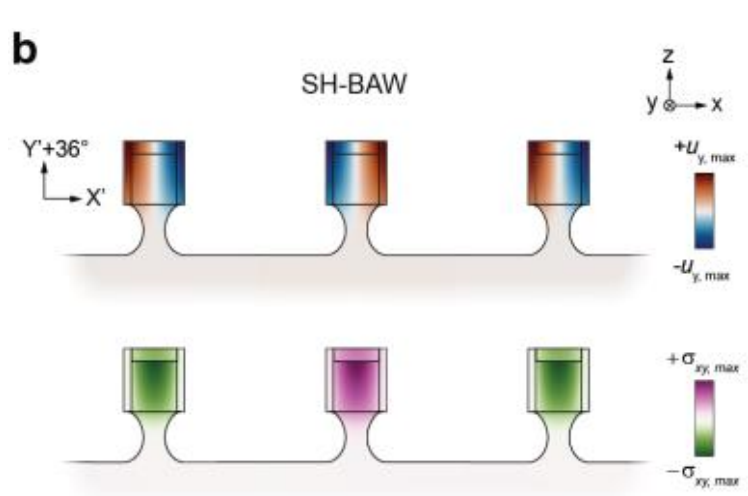

Prof. Guillermo Villanueva
Guillermo.Villanueva@epfl.ch
Ph.: +41 21 693 11 87
http://nems.epfl.ch

EPFL

Comment 2:

Electrodes are applied to the sidewalls of the $LiNbO_3$ ridges to excite in-plane propagating SH waves, as shown in Fig. S1. From the perspective of the crystal material, which piezoelectric coefficient is primarily utilized here? For example, in Z-cut LN, the e_{15} coefficient is typically used to excite the A_1 mode.

Response:

We thank the reviewer for this question. The resonator utilizes the e_{16} coefficient, which couples the horizontal field to the stress component with index 6 in Voigt notation, which is the shear horizontal stress field shown in Fig. 1b. For YX36°-cut, if the field is applied along the crystalline X axis, e_{16} takes a large maximum value roughly 4.4 C/m^2. We refrain from an in-depth discussion on this matter in the manuscript because the excitation of SH waves and piezoelectric coefficients for this specific cut have already been discussed in several prior works (for instance ref. 38, which we cite where the choice of crystalline cut is mentioned).

Changes:

- L102 & 137 of the revised version:

… we use YX36°-cut $LiNbO_3$ as a piezoelectric material, which possesses strong electromechanical coupling coefficients for shear acoustic waves for excitation with in-plane electric fields (e_{16})[38]

Due to the strong e_{16} piezoelectric coefficient in YX36°-cut $LiNbO_3$, our simulations predict k_t^2 …

Comment 3:

The author mentions in the text that in VBAR technology, "the pitch of the array does not influence the resonance frequency and can be chosen arbitrarily large to facilitate fabrication." While this simplifies lithography for the pitch, the critical dimension for frequency control—the w_{ridge}—remains extremely challenging, requiring lithography at ~600 nm, which poses significant fabrication demands. Currently, surface acoustic wave (SAW) technology based on LN/SiC can achieve frequencies around ~7 GHz while also offering a high keff2. In light of this, what does the author consider to be the key advantages of the VBAR approach when evaluated comprehensively?

Response:

We appreciate this remark and question. We must admit that the sentence that the reviewer mentions is vague, since "fabrication" can include many different processes. As the reviewer correctly points out, the critical dimensions (CDs) for lithography constrains the minimum width of the ridges and the pitch does not relax this constraint. However, we want to note that 600 nm is well within the capabilities of i-line or DUV stepper systems. The statement "to facilitate fabrication" is meant to refer to the etching and electrode deposition processes and not the lithography. The ability to choose an arbitrarily large pitch helps to increase selectivity and minimize non-uniformities at feature scale during the etching processes. Furthermore, the large openings enable the conformal deposition of the electrodes on the sidewalls. This would be much more difficult if the ridges were required to be separated by only a few hundred nanometers.

About frequency scaling, we would like to point out that for the VBARs, the CDs determine the minimum half wavelength ($\lambda/2$), while for SAW it is $\lambda/4$ (the width of the IDT electrodes assuming 0.5 metallization ratio). For instance, assuming a CD of 500 nm, the minimum λ of a SAW resonator would be 2 µm which typically results in frequencies < 2 GHz for the SH-SAW resonance with both LN/Si or LN/SiC (e.g. 10.1109/TMTT.2024.3383476 and 10.1109/JMEMS.2024.3369639), while VBARs with w_{ridge} = 500 nm resonate at 2.7 GHz. Nevertheless, we do not want to argue that VBARs have superior frequency scaling capability, since the Q of the devices we show becomes notably worse when going higher in frequency.

We believe that the main advantages are the following:

- k_t^2 for LN/SiC SAW reaches roughly 25% for optimized structures. The VBARs we show can reach a k_t^2 of more than 30% or in some cases even more than 35%.

Advanced NEMS Group
EPFL-STI-IGM NEMS
MED 2 2726, Station 9
CH-1015, Lausanne Switzerland

Prof. Guillermo Villanueva
Guillermo.Villanueva@epfl.ch
Ph.: +41 21 693 11 87
http://nems.epfl.ch

- Wafer cost: SAW resonators going above 3 GHz, and especially the demonstrations above 5 GHz using the LLSAW mode that the reviewer mentions, often rely on a SiC substrate. VBARs can be fabricated using cheaper LN/Si substrates.
- Footprint scaling: The capacitance in VBARs can be scaled by changing the height of the ridges. In contrast to SAW devices, the capacitance is not strictly tied to the footprint of the resonator which may be a useful property in some cases where constraints on device areas exist.

We want to mention here that the purpose of this manuscript is to demonstrate a rather unique, research-stage technology outside of the boundaries of common SAW or BAW architectures. We do not claim that it could or should replace any of these established commercial technologies in the near future.

Changes:

- L.121-123 in the revised version:

  Therefore, the pitch (p) of the array does not influence the resonance frequency and can be chosen arbitrarily large ~~to facilitate fabrication~~, which may facilitate the etching and electrode deposition processes required to fabricate the structure.

Comment 4:

Given the relatively low Q observed in the fabricated resonators, which may be attributed to factors such as sidewall roughness and cross sectional tilt angle of the ridges, has the sidewall roughness been characterized? Alternatively, have any comparative experiments with different etching angles been conducted?

Response:

We did not quantify the sidewall roughness (for instance with AFM) other than a qualitative visual assessment on the SEM images (Fig. 2b). Our simulations early on showed that the k_t^2 of the resonators decreases if the sidewalls are not vertical. Thus, we focused on developing a process that would yield vertical sidewall. We found that we can obtain sidewalls with 90° +/- 1° profile when using an IBE fixture angle of -45°.

As for comparative experiments with different etching angles, we *only* have non-piezoelectric test samples that we produced to assess the effect of the fixture angle on the sidewall angle. All the real samples with actual resonators were unfortunately fabricated using the "ideal" process that yields the most vertical sidewall angle. We agree that it would be interesting to try other angles and measure how the resulting non-vertical sidewall angle and possibly less/more roughness impacts the resonator.

Changes:

- We updated Fig. S7 in the SI with an additional image showing a clearer view of the etched $LiNbO_3$ sidewall.

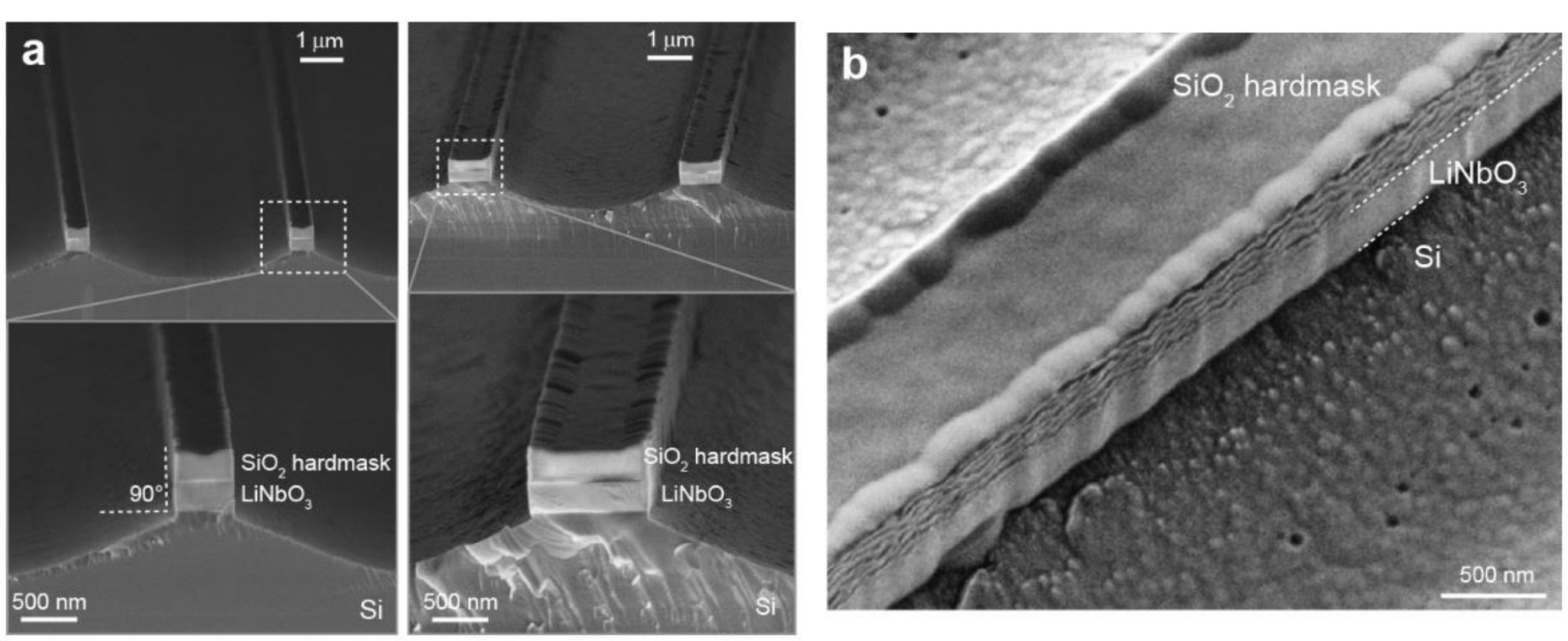

Advanced NEMS Group
EPFL-STI-IGM NEMS
MED 2 2726, Station 9
CH-1015, Lausanne Switzerland

Prof. Guillermo Villanueva
Guillermo.Villanueva@epfl.ch
Ph.: +41 21 693 11 87
http://nems.epfl.ch

Fig. S7 Details on the $LiNbO_3$ etching processes. a Cleaved test sample after IBE of a thin $LiNbO_3$ film with the wafer fixture set for an ion incidence angle of 45° and a SiO_2 hard mask. With this fixture angle, the etch rate is significantly faster in the center of large features, but as seen in the initial test with a dummy sample (Fig. S6), the sidewalls are vertical. **b** Tilted side view of the etched ridge showing the sidewalls of the SiO_2 hard mask and the underlying $LiNbO_3$.

Comment 5:

How is the width of Al electrodes controlled, and does this parameter significantly affect device performance?

Response:

We thank the reviewer for this question. The width of the Al electrode is controlled by the duration of the sputtering process. Preliminary tests showed that the deposition rate on the sidewalls is roughly 2/3 of the rate on a flat surface with our sputtering system.

The width of the electrode affects device performance in a similar manner as the thickness of the electrodes in an FBAR. This means that wider or heavier electrodes reduce the resonance frequency. If the electrodes make up a large enough fraction of the total width of the acoustic cavity, the k_t^2 is also going to be affected. We decided not to discuss this parameter in depth because we do not have experimental data of devices with different electrode widths. We focused on changing w_{ridge} and keeping the electrode width always the same.

Comment 6:

In Figure 2(c), the trace corresponding to a Wridge of 600 nm appears relatively faint and somewhat indistinct. If convenient, it would be helpful to slightly darken its color for better visual clarity.

Response:

We appreciate that the reviewer pointed out this oversight. We recolored Fig. 2c for better visual clarity.

Changes:

- Updated color scheme for Fig. 2c

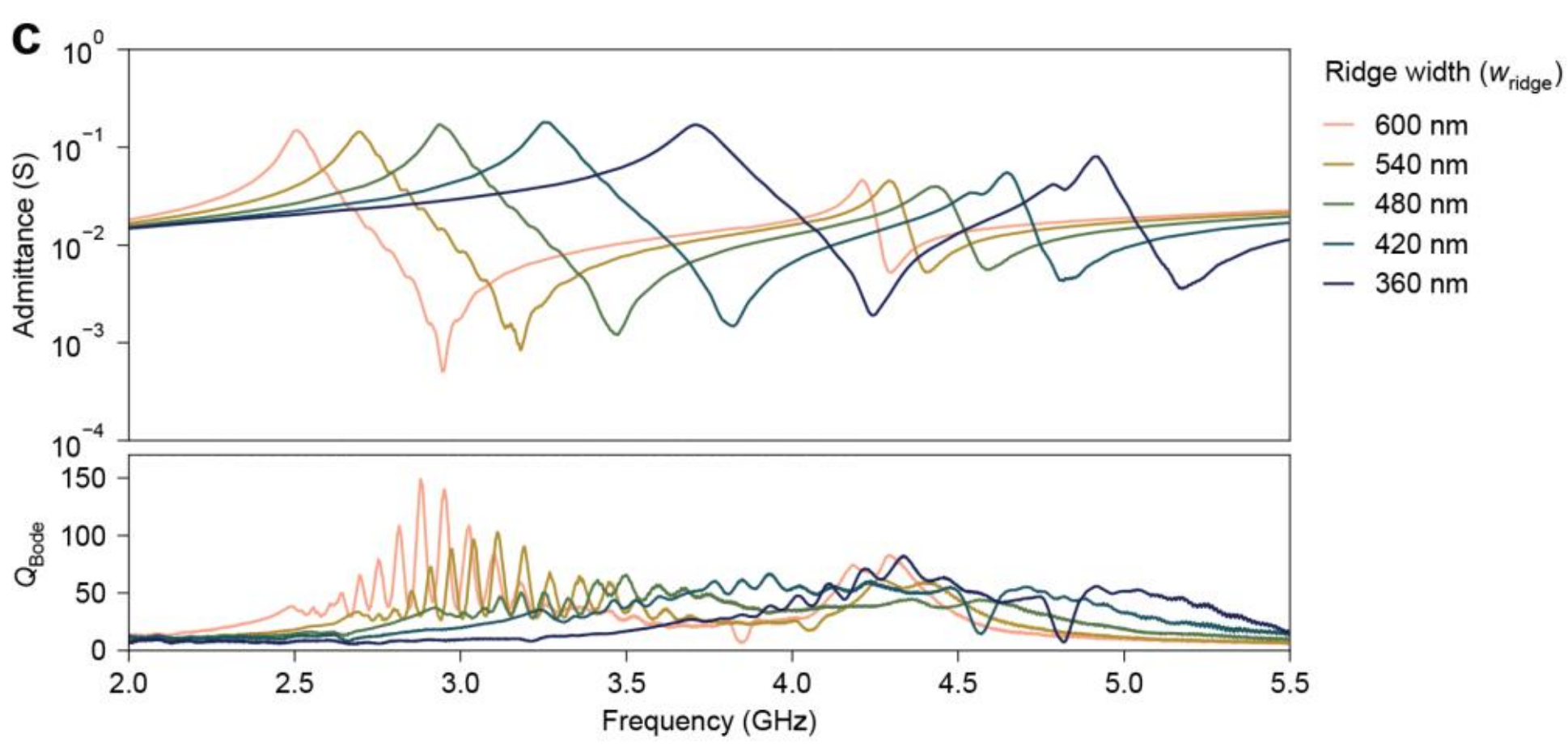

Advanced NEMS Group
EPFL-STI-IGM NEMS
MED 2 2726, Station 9
CH-1015, Lausanne Switzerland

Prof. Guillermo Villanueva
Guillermo.Villanueva@epfl.ch
Ph.: +41 21 693 11 87
http://nems.epfl.ch

Comment 7:

The author mentions in the text that "the static capacitance of a VBAR can be scaled by increasing the thicknesses of the $LiNbO_3$ and SiO_2 layers." What are the respective static capacitance values of the series and shunt resonators in the filter, and what are the corresponding device parameters? The paper does not provide a clear mapping between the static capacitance and the relevant device parameters.

Response:

We thank the reviewer for this comment. The resonators in the filters were designed to have static capacitances of 460 fF (series) and 810 fF (shunt). In the revised version, Fig. 4b and its legend now include the design parameters for the resonators of the three filters. Moreover, we have added a brief discussion what determines the static capacitance of our devices to the Supplementary Information, Section S2

Changes:

- Added dimensions to Fig. 3b and its legend

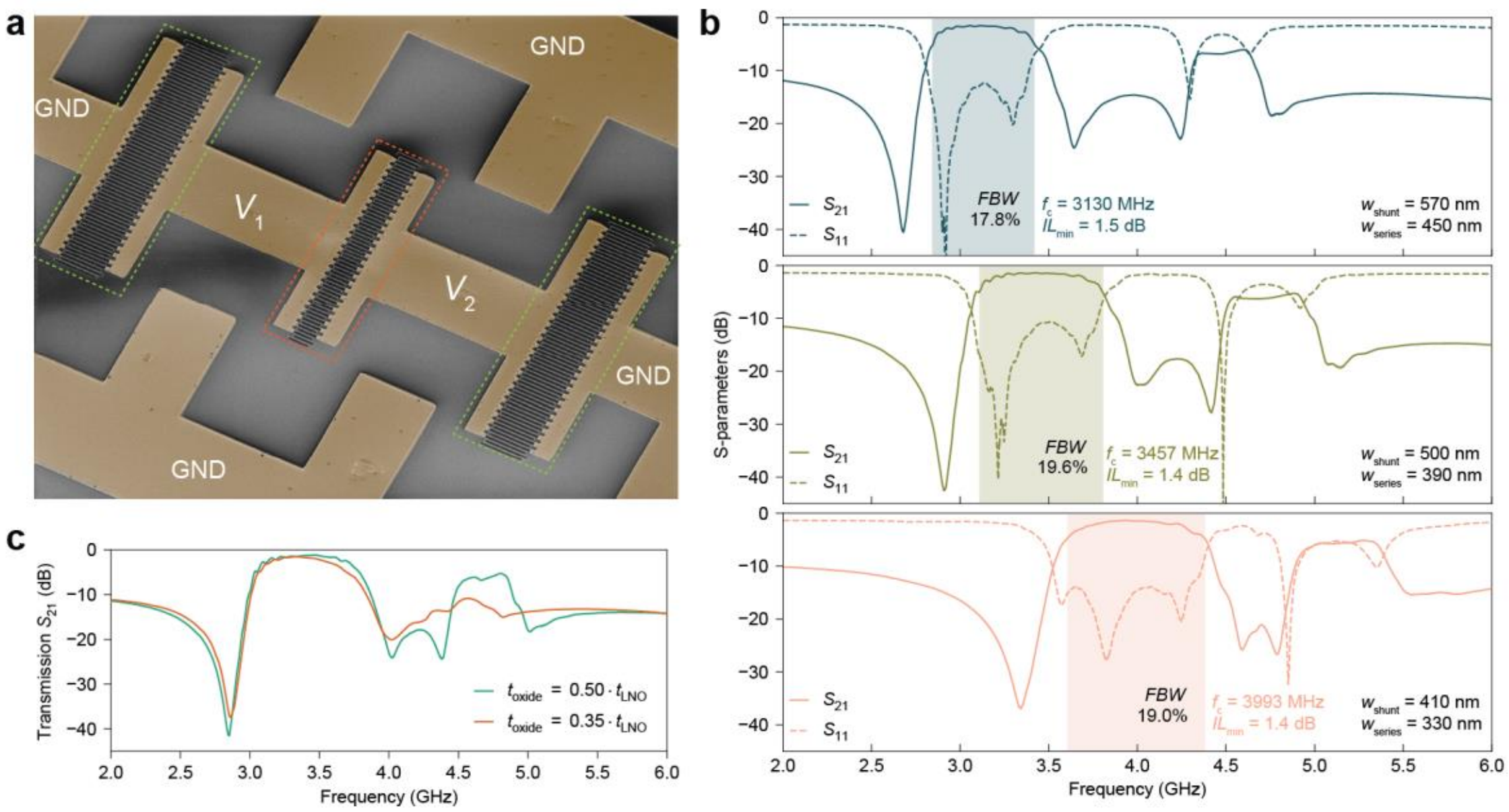

Fig. 4. Bandpass filters using VBAR building blocks. a False-color SEM images of a fabricated filter consisting of one series (red frame) and two shunt (green frame) VBARs. **b** Measured filter responses for three filters with different center frequencies (f_c). f_c is set by lithographically varying w_{ridge} of the series and shunt resonators (inset text). The aperture length (L) and number of ridges (N) of the resonators are adjusted to achieve a C_0 of approximately 810 fF (shunt) and 460 fF (series). The resulting dimensions are $L = 70 \cdot w_{ridge}$, $N = 60$ (shunt) and $L = 40 \cdot w_{ridge}$, $N = 50$ (series). For these three filters, $t_{oxide} = 0.5 \cdot t_{LNO}$. **c** S_{21} of identical filters with different t_{oxide}.

- We elaborated on the content in Fig. S3 in Section 2 of the Supplementary Information and added a brief discussion on C_0.

- L147 in the revised version:

Since the main SH-BAW mode propagates across the width of the ridge, the absolute thickness of the $LiNbO_3$ (t_{LNO}) is not the primary factor that determines f_r and k_t^2. The choice of t_{LNO} has a significant impact on C_0 and the appearance of thickness-confined spurious modes. ~~However, depending on the chosen thickness, thickness-dependent spurious modes might appear close to the main SH-BAW mode.~~ Further discussion and simulations that elucidate the impact of the layer thicknesses are given in the Supplementary Information, Section S2.

Advanced NEMS Group
EPFL-STI-IGM NEMS
MED 2 2726, Station 9
CH-1015, Lausanne Switzerland

Prof. Guillermo Villanueva
Guillermo.Villanueva@epfl.ch
Ph.: +41 21 693 11 87
http://nems.epfl.ch

Reviewer #2

The paper reports an impressive demonstration of novel GHz resonators in thin film lithium niobate. The most remarkable part is the inclusion of a filter demonstration. The thorough analysis is also impressive. The work will have a significant impact; therefore, it should be published after minor revisions.

Comment 1:

One major point is how the device will perform when it is fully suspended. The authors have compared solidly mounted and partially released. The main remaining uncertainty is how the Q, TCF, and IMD3 will change upon release. However, the reviewer recognizes that the authors may not wish to do so, given that the paper is still under review and that they may want to repurpose the device for other explorations. The reviewer suggests that the authors consider doing so at the end of the review process or expanding this discussion for a future conference publication. The paper should still be publishable, provided that a brief comment on this is included in the manuscript.

Response:

We would like to thank the reviewer for this suggestion. The partially released nature is one of the main ideas behind the resonator architecture, since we want to address the concerns about power handling and mechanical fragility that are commonly raised with regards to suspended resonators. We do not expect those resonators to perform very well when fully released as this was never intended. However, we agree that comparing with fully released resonators would be highly interesting to deepen our understanding of the physics and the loss mechanisms in the resonator.

We have released (XeF_2 gas) a subset of the resonators from the chip on which the measurements for Fig. 2h were taken and we have added the measured data of a fully released device to Fig. 2h. With the constraint of the Si gone, the resonance frequency drops and k_t^2 increases, which aligns qualitatively with the simulations of a fully released resonator which we have added to Fig. S2 in the Supplementary Information. In contrast to the simulation, the measured Q of a fully released resonator is interestingly significantly lower than the partially released (and intended) configuration (~30 vs. ~75). We believe that this could further strengthen our suspicion that the surface (e.g. roughness) is the problem in our devices and not bulk radiation through the anchor.

Changes:

- We added a measurement of a fully released resonator to Fig. 2h and updated the legend accordingly.

h

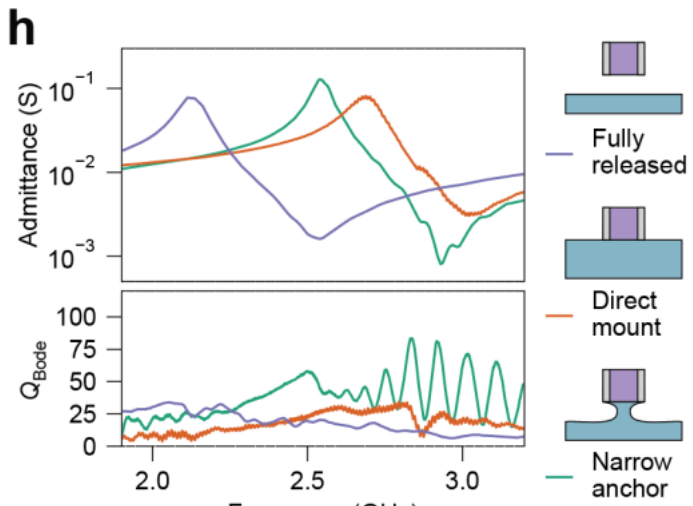

h Measured admittance of a VBAR (w_{ridge} = 600 nm) with a narrow anchor (as shown in **b**) compared to fully released and directly mounted devices.

- We rewrote the discussion about the losses to include the data that we added to Fig. 2h (L219-227 in the revised version).

…we fabricate a set of VBARs in which the ridge is directly mounted to the substrate ~~without the anchor~~or fully released (Fig. 2h). In the directly mounted configuration, the peak admittance is significantly lower compared to the standard VBAR with an anchor. As suggested by our simulations (see Fig. S2 in the Supplementary Information), the acoustic energy is poorly confined for the directly mounted configuration and therefore, the measured Q_{Bode} never exceeds 30~~, which indicates poor energy confinement~~. Even though fully releasing the ridge eliminates the possibility of substrate leakage, it also further increases the surface area. We observe a lower Q_{Bode} for the fully released resonators compared to the standard configuration

Advanced NEMS Group
EPFL-STI-IGM NEMS
MED 2 2726, Station 9
CH-1015, Lausanne Switzerland

Prof. Guillermo Villanueva
Guillermo.Villanueva@epfl.ch
Ph.: +41 21 693 11 87
http://nems.epfl.ch

with an anchor. This supports the notion that ~~radiation to the bulk is reduced by the narrow anchors~~ using a narrow anchor configuration, substrate leakage is largely suppressed and ~~that this loss mechanism is~~ not the predominant reason for the overall low Qs. The trends we observe point towards surface loss mechanisms as the limiting factor for Q.

- We updated Fig. S2 with simulated data of a fully released ridge

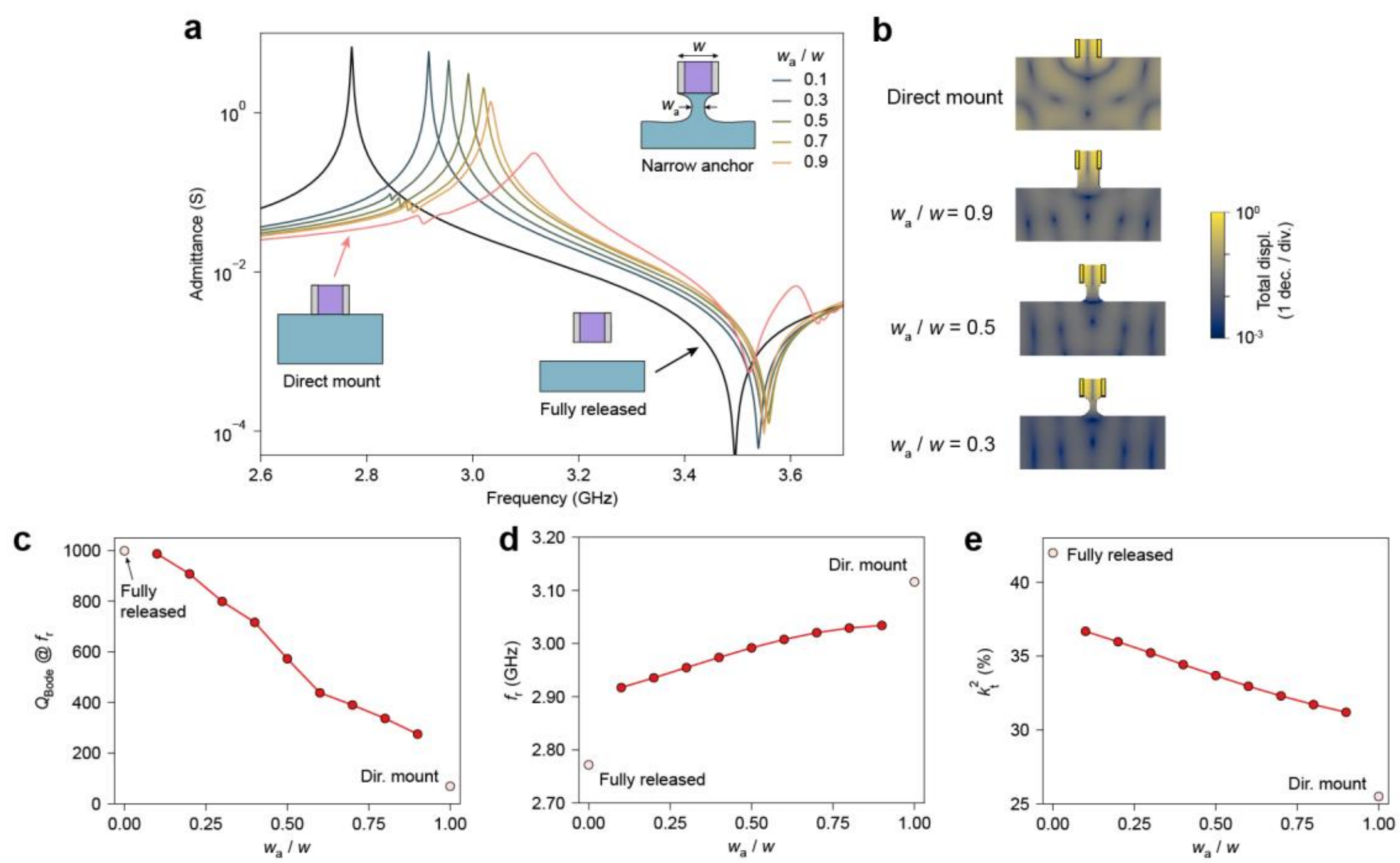

Comment 2:

The IMD3 measurement should be better performed for the filter, as it is less measurable or comparable for resonators, due to 1) the high reflection nature and 2) the dramatic impedance contrast of the resonator. The IMD3 filter is a better measure of the matrix. The two-port IMD3 measurement also aligns more closely with the definition. The authors should consider reporting that instead. However, as noted in the previous comment, the paper is publishable without this; it would be strengthened.

Response:

We thank the reviewer for this comment. We agree with the reviewer's concerns and opinions regarding IMD3 measurements of single resonators. We would have also preferred to perform these measurements on the filters. Unfortunately, the hardware components in the setup we used for the IMD3 measurements limited us to input tones of maximum 2.7 GHz, which is lower than the passband of the lowest-frequency filter we fabricated. We changed the sentence that refers to the IMD3 measurement and figure legend of Fig. 3f to make sure that readers are aware of the particularities of the measurement configuration. We are updating our measurement setup and fabricating new samples and in a few months we will have more data on filters.

Changes:

- L256-258 in the revised version:

  In addition, using a two-tone ~~setup~~ excitation in a one-port reflection configuration, we measure the third-order intermodulation distortion (IMD3) ~~induced by the non-linear effects in the resonator~~ in the reflected signal arising from nonlinearities of the resonator (Fig. 3f).

- Legend for Fig. 3f in the revised version

  f *One-port measurement of the IMD3 in the reflected signal ~~IMD3 measurement~~ of a VBAR…*

Comment 3:

Could the authors comment on why the SH0 mode, but not the S0/A1/S1 mode are used here? The reviewer assumes that A1/S1 might not be confined, but that S0 might be at some risk? That would make the frequency even higher.

Response:

We thank the reviewer for this interesting question. Yes, we use the SH0 mode because it can be confined and excited with high k_t^2. In contrast to continuous suspended films, the acoustic cavity here is also confined by vertical free boundaries. We wanted to have a wave that is not prone to mode conversion when reflecting on the vertical free boundaries. The SH wave (with displacement parallel to the boundaries) reflects and transmits only as an SH wave and thus the acoustic energy can be confined if the anchor does not support the transmission of an SH wave (as detailed in the SI, Section 1). For the other modes, the displacements are mainly in the sagittal plane (along x and z in Fig. 1b), which can generally generate waves in the anchor that can transmit into the substrate. We have seen in simulations that all other modes are accompanied by significant leakage due to this, especially when the width and thickness of the ridge are on the same order of magnitude. It may still be possible to find specific combinations of dimensions that limit this phenomenon. For example, we have seen in our simulations that the equivalent of the S0 mode (i.e. a horizontally propagating longitudinal BAW) can achieve reasonable Q if the thickness of the ridge ($LiNbO_3$ film) is much larger than the width of the ridge (i.e. 10 µm thickness with w_{ridge} = 500 nm).

Comment 4:

The side wall might be the bottleneck here. Have the authors considered alternative metallization methods? The reviewer recalls ALD used for those HZO finbar devices. Would that be applicable here?

Response:

We are grateful for the reviewer's suggestion. We also suspect that the sidewall is one of the major limitations. We have considered ALD from the beginning and believe that it would be a better option than sputtering for the electrode deposition. Unfortunately, at the time of the fabrication of these devices, none of the ALD tools in our cleanroom offered the possibility to deposit a suitable metal. Thus, we resorted to sputtering as an alternative at the time, but we are moving to ALD in the future.

Comment 5:

Good to comment on how to develop further the technology, e.g., the intrinsic challenges of the structure and potential ways to overcome those.

Response:

We thank the reviewer for this suggestion. We wanted to discuss this in the last section, but we realize that the initial version of the manuscript lacked clarity on these points. We have rewritten parts of the Discussion section to clearly communicate what we believe the intrinsic challenges are.

Changes:

- In the Discussion:

…It is probable that the power handling could be improved if the losses and thus heat generation in the resonators are minimized.
Several intrinsic challenges of the vertical geometry must be addressed to further develop the technology. The large surface-to-volume ratio of the present thin-film implementation increases the sensitivity to surface roughness or other fabrication imperfections. From a design perspective, using thicker $LiNbO_3$ films on the order of a micron or more may be beneficial for VBARs. The lower surface-to-volume ratio may weaken the detrimental influence of a lossy surface, and the influence of any non-uniformities in the

anchor dimensions on the resonance frequency would also be reduced. In any case, further optimization of the fabrication process aimed at lowering the roughness of the interfaces and electrode sidewalls is desirable. An example of such an improvement could be to replace sputtering for the electrode metal with atomic layer deposition (ALD). The implementation of VBARs with substantially thicker $LiNbO_3$ introduces the additional challenge of deeper $LiNbO_3$ etching. Addressing this will require further process development to establish an etching method that not only achieves the necessary depth and mask selectivity but also maintains smooth and vertical sidewalls.

Provided these challenges can be successfully addressed, the concept of a non-planar resonator offers a combination of functionalities that cannot be readily achieved with conventional planar structures. We have shown that such a structure supports BAW resonances with higher k_t^2 than SAW resonances in $LiNbO_3$, while also providing lithographic frequency control similar to SAW devices, which enables relatively straightforward implementation of filter prototypes. Furthermore, the general idea of a vertical, non-planar BAW resonator implies…

Comment 6:

The fabrication process is quite impressive. Could the authors include more images of the devices at the intermediate fabrication steps in the supplementary document? The reviewer is interested in seeing the entire process and assumes readers will be as well.

Response:

We appreciate the reviewer's interest in our fabrication process. We are showing some additional SEM images in the revised Supplementary information. A lot of the fabricated features are only recognizable in a cross-sectional view which requires the samples to be cleaved, rendering them unusable. Hence, we only have comprehensive images of test samples and not the actual prototype samples. We hope that the reviewer may still find interest in those. We have combined them with the images shown in the initial version.

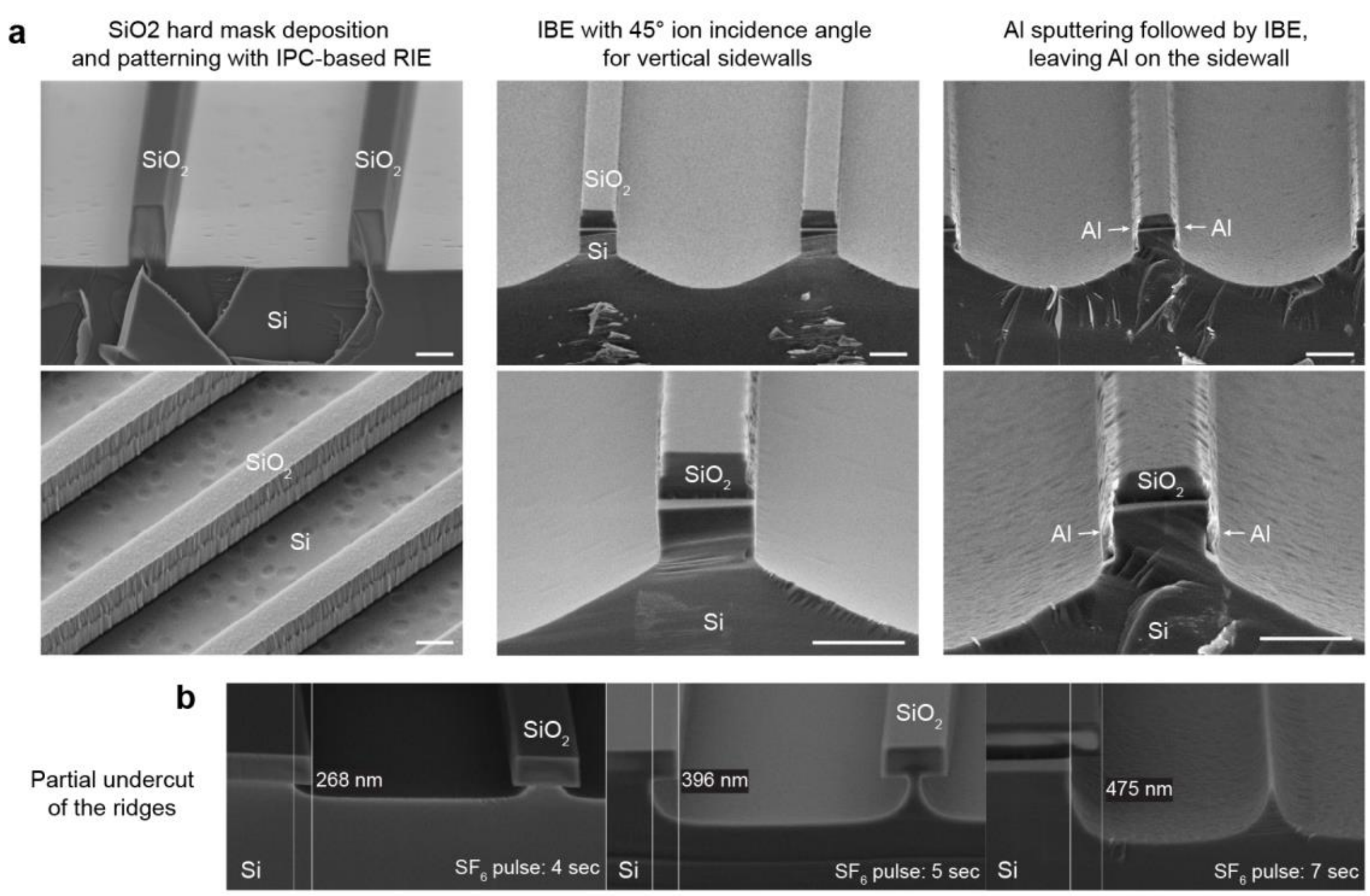

Fig. S6 Process development on dummy samples. a Main process steps to fabricate the ridges on a dummy Si sample without a $LiNbO_3$ layer. **b** Test sample (patterned SiO_2 ridges on Si wafer, no sidewall electrodes) to showcase the dry etching process (ICP SF_6 with a low frequency wafer voltage bias) for the partial release of the ridges. The width of the Si pedestal under the ridge can be controlled with etch duration. The recipe is

designed for deep anisotropic etching of silicon but features a consistent undercut that correlates with the duration of the etch pulse. If the pulse is too long (7 sec.), the undercut is too large, and the ridge is fully released.

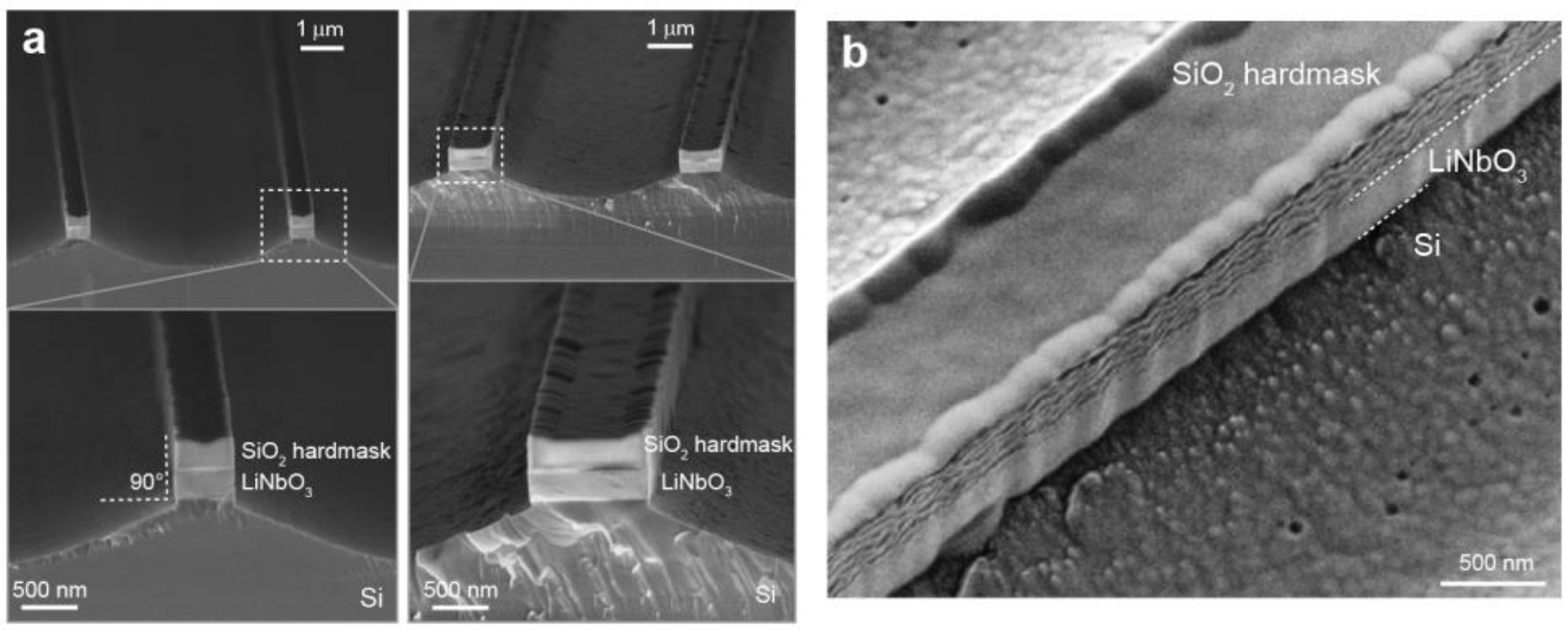

Fig. S7 Details on the $LiNbO_3$ etching processes. a Cleaved test sample after IBE of a thin $LiNbO_3$ film with the wafer fixture set for an ion incidence angle of 45° and a SiO_2 hard mask. With this fixture angle, the etch rate is significantly faster in the center of large features, but as seen in the initial test with a dummy sample (Fig. S6), the sidewalls are vertical. **b** Tilted side view of the etched ridge showing the sidewalls of the SiO_2 hard mask and the underlying $LiNbO_3$.

Reviewer #3

It is an interesting work. I have several questions and comments:

Comment 1:

For power handling measurement (Fig. 3d), what is the actual power going through the resonator? What is the power density? It is better to add the SEM images of the damaged resonator after the power test.

Response:

We thank the reviewer for the question and the suggestion. The values on horizontal axis in Fig. 3d are the incident power at the RF probe which is in contact with the resonator (and not simply the input of the setup). Since the insertion loss and of the probe is small, these values correspond approximately to the power driving the resonator. At the frequency of the measurement, S_{11} of the resonator is approximately -4.5 dB which corresponds to ~ 0.6 in linear scale. From this, we estimate that for an input power of 30 dBm (1 W), roughly $P_{abs} = 1\ \text{W}\cdot(1 - 0.6^2) = 0.64$ W are absorbed and dissipated in the resonator. The total volume of the ridges which is ~ 50 · 42 um · 500 nm · 750 nm ≈ $7.9\cdot10^{-16}$ m^3. We do not think that giving a number on power density is meaningful, since we do not know exactly how the dissipated power is spatially distributed. Plus, such values are not typically reported in the context of acoustic resonators. We added a SEM image of the damaged resonator after the power test to Fig. 3.

Changes:

- Added an additional panel to Fig. 3 showing SEM images of the damaged resonator.

e

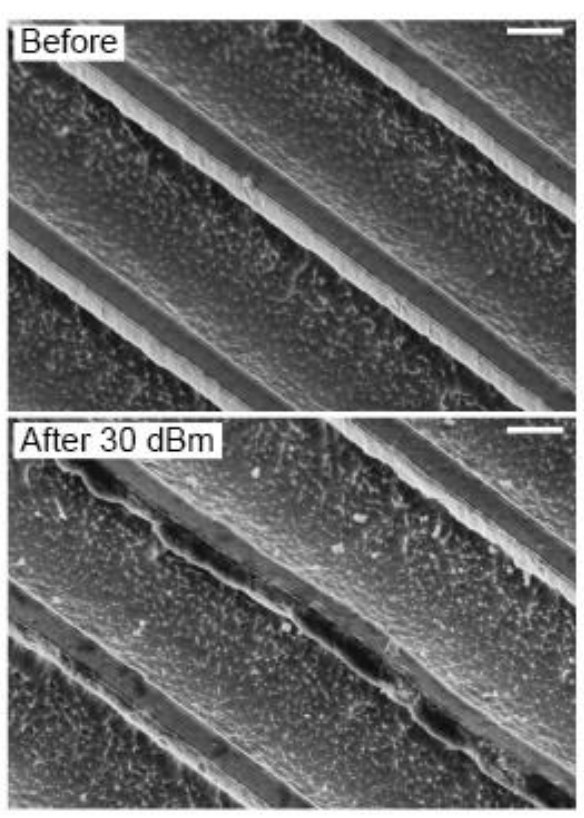

e SEM images of a VBAR before and after subjecting the device to a 30 dBm tone. Scale bar 1 µm**.**

Comment 2:

The frequency uniformity of each pair is good. How to achieve it? Is this due to good fabrication, or is there coupling between each pair in one resonator?

Response:

We are grateful for this question. The acoustic mode is confined within each ridge and thus there should not be any coupling between adjacent ridges. The layout of the resonators is deliberately designed to improve the uniformity of the different processes. For example, all the openings where we etch to define the width of the ridge are the same size for all devices to ensure that the etch rate is the same everywhere. Furthermore, even though it would not be strictly needed for the dimensions we show, we use E-beam lithography for this mask. We think that this certainly helps in ensuring that the ridge width is uniform. However, there may still be some non-uniformities which are hard to quantify, i.e. the width of the Si anchor. It is possible that the effect of this is masked by the rather low Q of the resonance. Once we improve the Q, the effect of these non-uniformities may become more visible.

Advanced NEMS Group
EPFL-STI-IGM NEMS
MED 2 2726, Station 9
CH-1015, Lausanne Switzerland

Prof. Guillermo Villanueva
Guillermo.Villanueva@epfl.ch
Ph.: +41 21 693 11 87
http://nems.epfl.ch

Comment 3:

How about TCF and the power handling of the filter?

Response:

We appreciate this question and are sorry for not showing these measurements in the manuscript. We have unfortunately not measured this on the filters. Considering that the filters only have a single series resonator, we estimate that the filter could survive *at most* the maximum power level that the resonator alone can withstand, that is 29-30 dBm.

Comment 4:

Do you have the data to support the effect of the size of the Si anchor on the quality factor?

Response:

We thank the reviewer for this question. In Section 1 of the SI (Fig. S2), we show simulated data for the effect of the Si anchor size on the quality factor. Furthermore, in Fig. 2h we show experimental data that compares configurations with and without a Si anchor. Experiments with resonators with different anchor widths would take months since one different sample must be procured per width.

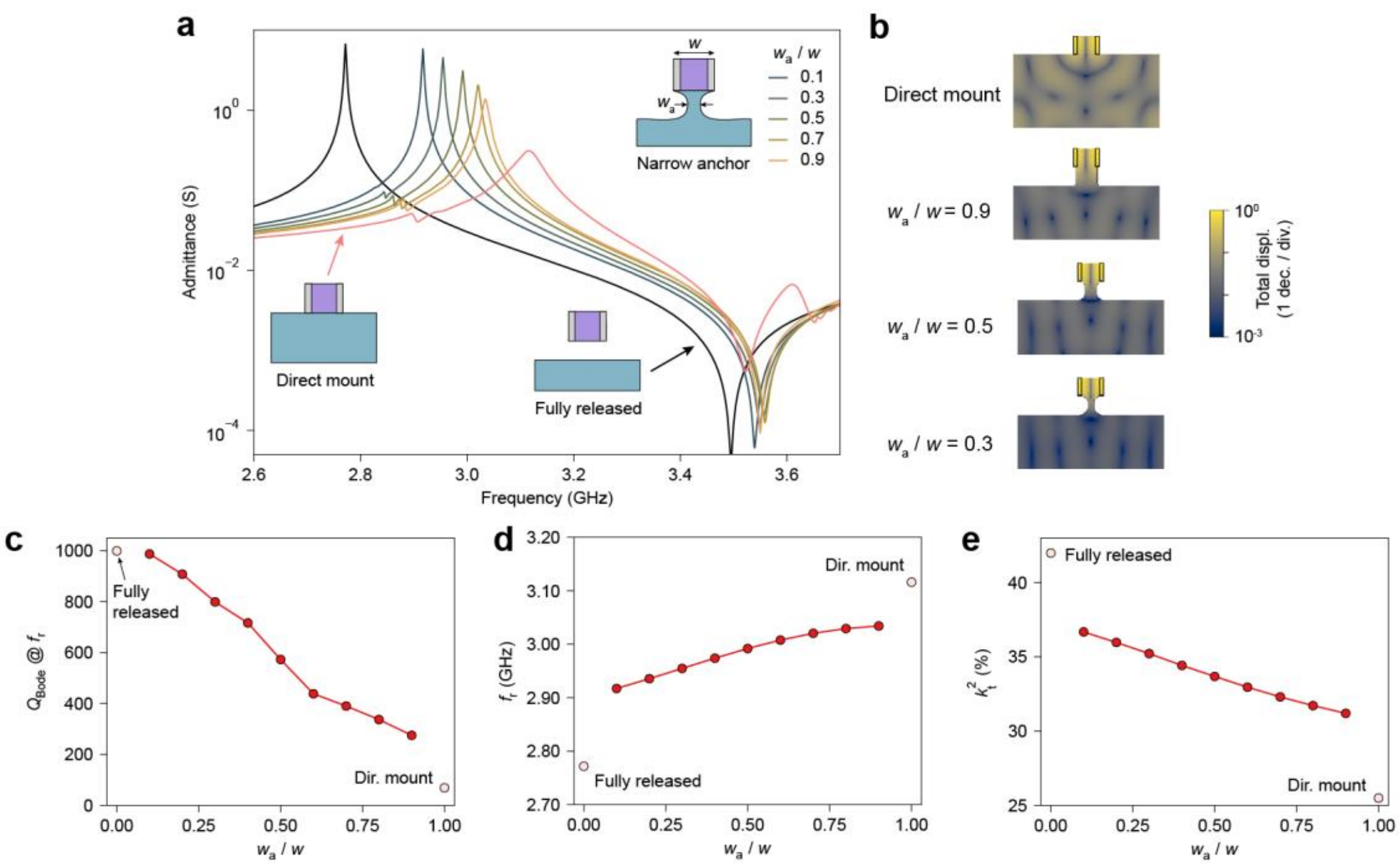

Fig. S2 Simulated admittance and eigenmodes for different anchor dimensions. a Simulated admittance for a $LiNbO_3$ ridge with three different mounting configurations: mounted directly to the silicon substrate (no anchor), mounted with an anchor with a width w_a at the narrowest point, or fully released. **b** Eigenmodes corresponding to the admittance peak for different anchor configurations. The displacement is plotted with a logarithmic normalization to emphasize the presence of BAWs in the substrate. **c-e** Simulated **c** Q at resonance frequency (f_r), **e** f_r, and **f** k_t^2 as a function of w_a/w. For the simulations in this figure, the other dimensions of the resonators are w_{ridge} = 500 nm, w_e = 100 nm, t_{LNO} = 400 nm, t_{oxide} = 100 nm. w denotes the total width of the acoustic cavity ($LiNbO_3$ ridge plus twice the electrode width, $w_{ridge} + 2 \cdot w_e$). The intrinsic mechanical Q assigned to all materials (imaginary part of stiffness coefficients) is 1000.

Advanced NEMS Group
EPFL-STI-IGM NEMS
MED 2 2726, Station 9
CH-1015, Lausanne Switzerland

Prof. Guillermo Villanueva
Guillermo.Villanueva@epfl.ch
Ph.: +41 21 693 11 87
http://nems.epfl.ch

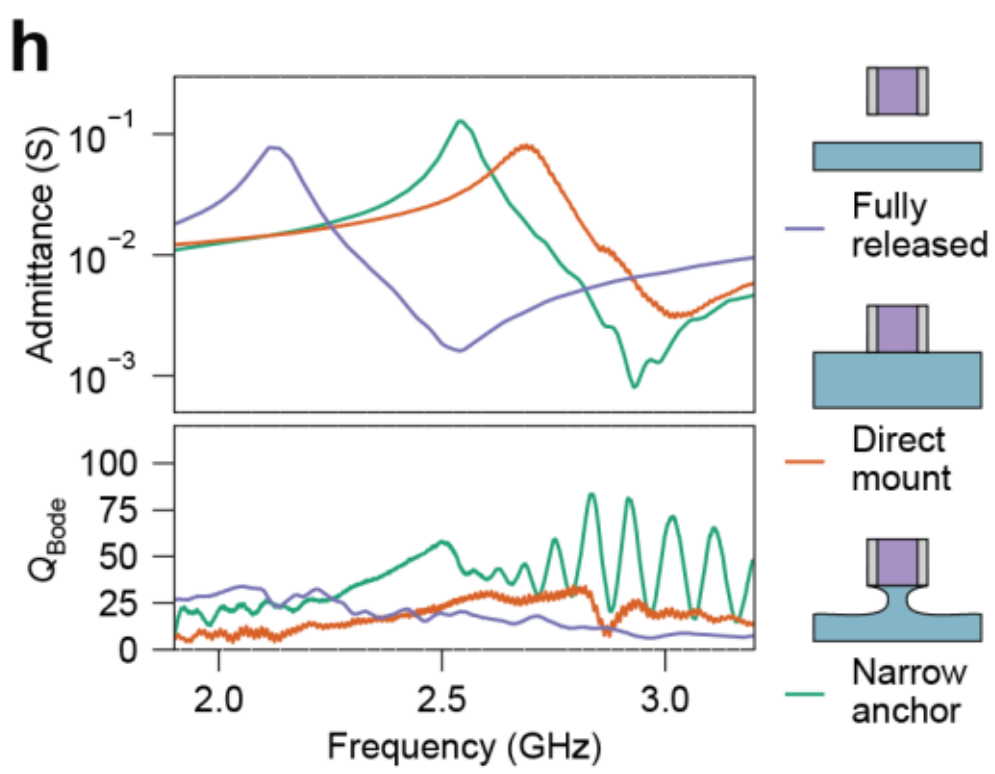

h Measured admittance of a VBAR (w_{ridge} = 600 nm) with a narrow anchor (as shown in **b**) compared to fully released and directly mounted devices.

Comment 5:

What is the limitation in improving the selectivity of the filter?

Response:

We thank the reviewer for this question. We think that there are two paths towards improving selectivity. The strongest limitation currently is the low Q of the resonator. We believe that optimizing the resonator for better Q could yield a noticeable improvement in selectivity even with the same filter design/topology. The second aspect that should be considered is the filter order. The topology of the filters that we show is quite basic and selectivity may be improved by, for instance, adding more ladder stages. We have not explored this option so far because the insertion loss would likely increase significantly due to the currently limited Q of the resonator.

Advanced NEMS Group
EPFL-STI-IGM NEMS
MED 2 2726, Station 9
CH-1015, Lausanne Switzerland

Prof. Guillermo Villanueva
Guillermo.Villanueva@epfl.ch
Ph.: +41 21 693 11 87
http://nems.epfl.ch

Manuscript Number: COMMSENG-25-0857A

Title: A bulk acoustic resonator with vertical electrodes for wideband filters

Authors: S. Stettler, E. Navarro-Gesse, C. Collado, J. Mateu, and L. G. Villanueva

April 27th, 2026

Dear Editor and Referees,

Thank you for your continued time and effort in reviewing our manuscript. Below, we have addressed the remaining questions from the second round of review and modified the text accordingly. We believe these latest revisions have further strengthened the manuscript, and we are grateful to the reviewers for their feedback throughout this process.

Legend: Reviewer's comments are typed in black over a gray background. Our responses are typed in black without any background shading. Changes are highlighted with a red font color. The line numbers refer to the clean revised version of the manuscript (without changes marked-up).

Reviewer #1

I thank the authors for their detailed rebuttal and improvements to the manuscript. My previous comments on crystallographic orientation, piezoelectric coefficients, and capacitance mapping have been adequately addressed, clarifying the VBAR's operating principles. I recommend publication after a few minor revisions to further contextualize the device's current limitations.

Comment 1:

The hypothesis that surface roughness limits the quality factor is reasonable but lacks quantitative verification, such as via AFM. Please add a brief statement in the manuscript explicitly acknowledging this limitation and clarifying that attributing low Q to surface losses remains a hypothesis for future study.

Response:

We thank the reviewer for this comment on the discussion of the surface roughness. We agree that the data we present is insufficient to confirm that the surface roughness is indeed the cause for the low Q. We have slightly reformulated the passage where we discuss the measured Qs and added a statement that clarifies that the impact of the surface roughness remains a hypothesis.

Changes:

- L.212 – L.219 of the revised version:

  This trend suggests that the high losses could originate from surface loss mechanisms such as surface roughness, which are a key factor determining Q in thin film $LiNbO_3$ resonators[42] and nanoelectromechanical resonators in general[43].This is supported qualitatively by the sidewall roughness that is visible in the SEM micrographs (Fig. 2b), as well as the high surface-to-volume ratio inherent to the nearly square cross-section of the ridges. However, we currently lack a quantitative measurement (i.e. AFM) of the roughness of the sidewalls. Thus, attributing the limited Q primarily to surface roughness remains a hypothesis for future studies. ~~This explanation also seems probable considering that the sidewalls are visibly rough. Further, the cross-section of the ridges is close to a square, which results in a significantly higher surface-to-volume ratio than a suspended film or a much taller ridge with the same width.~~ At f_r, the Q is further decreased…"

- L. 231 of the revised version:

Advanced NEMS Group
EPFL-STI-IGM NEMS
MED 2 2726, Station 9
CH-1015, Lausanne Switzerland

Prof. Guillermo Villanueva
Guillermo.Villanueva@epfl.ch
Ph.: +41 21 693 11 87
http://nems.epfl.ch

This supports the notion that using a narrow anchor configuration, substrate leakage is largely suppressed and not the predominant reason for the overall low Qs. ~~*The trends we observe point towards surface loss mechanisms as the limiting factor for Q.*~~

Comment 2:

Although experimental data for varying electrode widths is currently absent , it is a fundamental parameter that affects performance. Please add a brief theoretical discussion or simulated trend explaining how electrode thickness variations are expected to influence on f and Kt

Response:

We thank the reviewer for highlighting the importance of the electrode width. We have added a simulated trend and a short discussion on the electrode width to Section 2 to of the SI.

Changes:

- Addition of simulations and a short discussion on the electrode width to Section 2 of the SI.

The role of the electrode width (w_e) in a VBAR can be understood in a similar manner as the role of the electrode thickness in a conventional FBAR[2]. Fig. S3a shows the simulated admittance response of a VBAR with varying aluminum electrode width for a fixed ridge width w_{ridge} = 500 nm. Widening the electrodes represents an increase in the total width of the acoustic cavity and leads to a reduction in resonance frequency. For $w_e > 0.15 \cdot w_{ridge}$, a significant part of the total elastic energy starts to be concentrated in the electrodes instead of the $LiNbO_3$ which decreases k_t^2. Based on only the trends of f_r and k_t^2 for an Al electrode, w_e is ideally chosen to be $< 0.15 \cdot w_{ridge}$. However, thinner electrodes also increase the resistive losses of the resonator. Considering this trade-off, we target an electrode width of around 75 nm which corresponds to $0.15 \cdot w_{ridge}$ for VBARs with w_{ridge} = 500 nm. We note that while w_{ridge} can be adjusted lithographically for each individual device, the absolute electrode width (i.e. 70 nm for the fabricated devices) is controlled by the duration of the Al deposition and thus the same for all devices (independent of w_{ridge}) on a given sample.

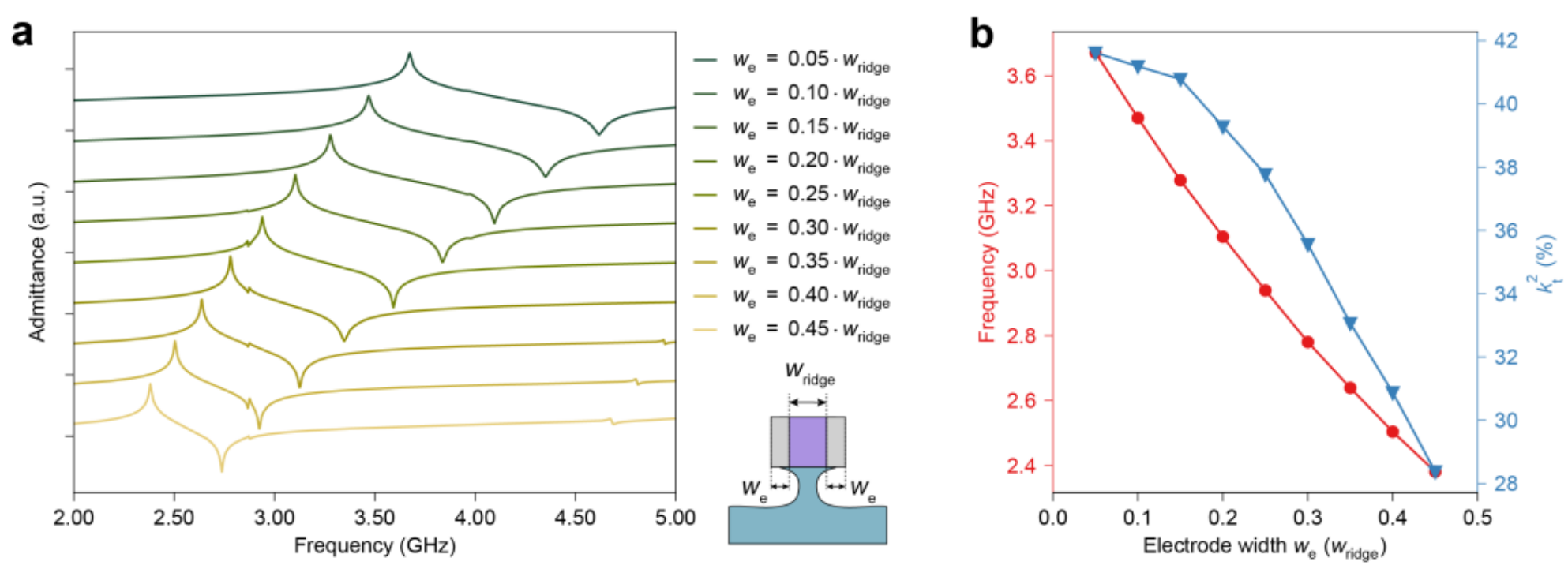

Fig. S3 Simulated dependence of the VBAR response on the width of the Al electrodes (w_e). a Admittance for varying electrode width. **b** Extracted resonance frequencies and k_t^2 as a function of w_e. For these simulations, w_{ridge} = 500 nm and t_{LNO} = 400 nm.

- L.147 of the revised version:

The electrode width (w_e) contributes to the total width of the acoustic cavity and thus affects the achievable f_r and k_t^2 for a given ridge width. Further discussion and simulations that elucidate the impact of the layer thicknesses and electrode width are given in the Supplementary Information, Section S2.

Advanced NEMS Group
EPFL-STI-IGM NEMS
MED 2 2726, Station 9
CH-1015, Lausanne Switzerland

Prof. Guillermo Villanueva
Guillermo.Villanueva@epfl.ch
Ph.: +41 21 693 11 87
http://nems.epfl.ch

Reviewer #2

The paper is well edited and should be published. The paper is a good innovation in the area.

Reviewer #3

The authors have answered most of my questions in the response letter, but they did not include the responses in their manuscript to improve clarity. Please allow me to repeat the question:

Comment 1:

For power handling measurement (Fig. 3d), what is the actual power going through the resonator? What is the power density? It is better to add the SEM images of the damaged resonator after the power test. *need to add the corresponding information to the manuscript*.

Response:

We thank the reviewer for their time in reviewing our paper and giving us suggestions. We did not understand that we had to add this information to the manuscript, but rather that the reviewer wanted clarifications on our work, which we think we provided. In addition, we did add an SEM image of the damaged resonator as Fig. 2e. For the power dissipated in the resonator, we thought that it was clear from Fig. 3c where we show S_{11} of the resonator, from which estimating the dissipated power is straightforward. This time, we have added this information explicitly in the text to avoid confusion. However, as mentioned in the previous revision round, we do not know exactly how the dissipated power is spatially distributed in the resonator, which makes it impossible to estimate the power density in a meaningful way. Hence, we cannot in good faith add a number to the manuscript.

Changes:

- L. 260 of the revised version:

  For incident power levels approaching 30 dBm (1 W, corresponding approximately to 0.6 W dissipated in the resonator based on the S_{11} at f_0, see Fig. 3c), the Al electrodes show signs of melting ~~appear to melt~~, and the ridges and ~~the ridges do not appear to be straight anymore~~ the ridges visibly deform.

e

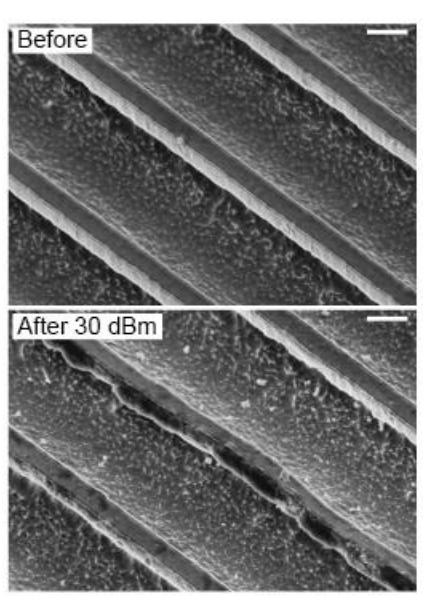

e SEM images of a VBAR before and after subjecting the device to a 30 dBm tone. Scale bar 1 µm.

Advanced NEMS Group
EPFL-STI-IGM NEMS
MED 2 2726, Station 9
CH-1015, Lausanne Switzerland

Prof. Guillermo Villanueva
Guillermo.Villanueva@epfl.ch
Ph.: +41 21 693 11 87
http://nems.epfl.ch

Comment 2:

The frequency uniformity of each pair is good. How to achieve it? Is this due to good fabrication, or is there coupling between each pair in one resonator? *need to add the corresponding information to the manuscript*

Response:

We thank the reviewer for their time in reviewing our paper and giving us suggestions. We did not understand that we had to add this information to the manuscript, but rather that the reviewer wanted clarifications on our work, which we think we provided.

We can know that the acoustic modes are quite confined within the ridges and thus the acoustic coupling between two ridges can be neglected.

To be more clear, let us say that we cannot measure the resonant response of single ridges. We can only access the collective response. Even though the frequency uniformity may appear good from the collective response, we cannot quantify how uniform these frequencies truly are. To try and add some extra information, we have added some clarifications on some of the processing and design choices that we selected to promote good dimensional uniformity of the ridges.

Changes:

- L. 352 of the revised version:

  We pattern the deposited SiO_2 layer using e-beam lithography, followed by an inductively coupled plasma (ICP) based dry etching using a $C_4F_8/H_2/He$ chemistry (SPTS Advanced Plasma System). Since the linewidth of the patterned SiO_2 features subsequently translates into the width of the $LiNbO_3$ features (i.e. the ridge width w_{ridge}), we use e-beam lithography for this mask to ensure high uniformity.

- In the legend of Fig. S7 in the SI:

  The recipe is designed for deep anisotropic etching of silicon but features a consistent undercut that correlates with the duration of the etch pulse. If the pulse is too long (7 sec.), the undercut is too large, and the ridge is fully released. The etch rate of this process also depends on the exposed area between two ridges. Thus, the spacing between ridges is kept the same for all fabricated devices to maximize the uniformity of the etch rate.

Comment 3:

How about TCF and the power handling of the filter? *need to mention in the manuscript why these data were not measured*

Response:

We thank the reviewer for their time in reviewing our paper and giving us suggestions. We did not understand that we had to add this information to the manuscript, but rather that the reviewer wanted clarifications on our work, which we think we provided. Indeed, measuring these parameters was originally not part of the idea of this paper which was supposed to remain mostly a paper showing a novel architecture of resonator, and opening the field for further measurements and research.

Even though we thought that these measurements were not necessary to validate publication of our paper, we have measured both TCF and the power handling of the filter in the meantime. We have added this data to Fig. 4 of the revised version because we believe it complements the data from the single resonators well. At the same time, we'd like to note that anything measured on the filter is always dependent on the topology of the filter (which is not the main point of this work) in addition to the properties of the resonator.

Advanced NEMS Group
EPFL-STI-IGM NEMS
MED 2 2726, Station 9
CH-1015, Lausanne Switzerland

Prof. Guillermo Villanueva
Guillermo.Villanueva@epfl.ch
Ph.: +41 21 693 11 87
http://nems.epfl.ch

As mentioned in our answer to another reviewer in the previous round, we cannot measure the non-linearities on the filters due to the frequency limitations of the hardware in the setup we use for the IMD3 and IP3 measurements.

Changes:

- Updated Fig. 4 with measured TCF and high-power characterization data of the filter

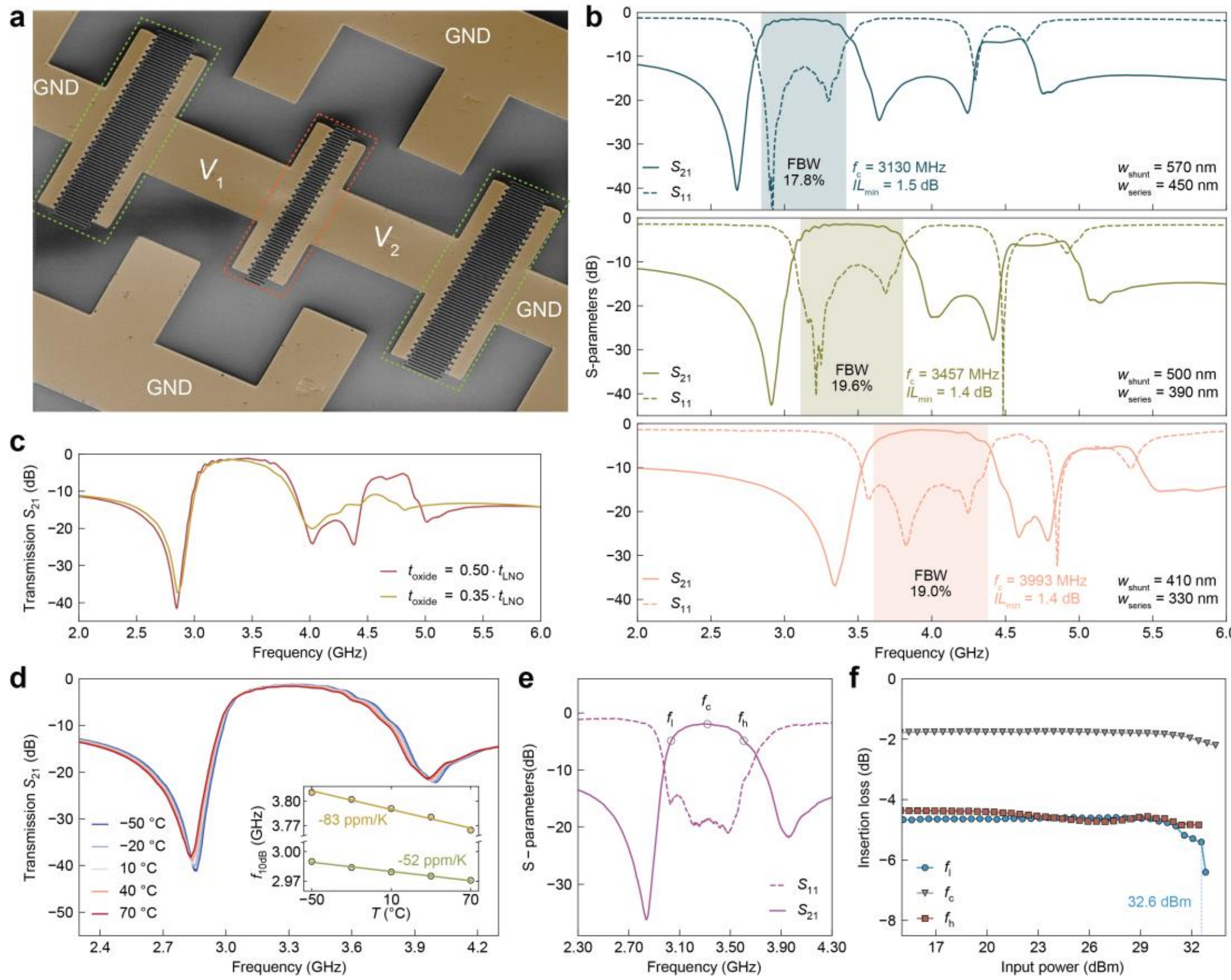

Fig. 4. Bandpass filters using VBAR building blocks. a False-color SEM images of a fabricated filter consisting of one series (red frame) and two shunt (green frame) VBARs. **b** Measured filter responses for three filters with different center frequencies (f_c). f_c is set by lithographically varying w_{ridge} of the series and shunt resonators (inset text). The aperture length (L) and number of ridges (N) of the resonators are adjusted to achieve a C_0 of approximately 810 fF (shunt) and 460 fF (series). The resulting dimensions are $L = 70 \cdot w_{ridge}$, $N = 60$ (shunt) and $L = 40 \cdot w_{ridge}$, $N = 50$ (series). For these three filters, $t_{oxide} = 0.5 \cdot t_{LNO}$. **c** S_{21} of identical filters with different t_{oxide}. **d** Change in S_{21} with temperature and extracted TCF of the 10 dB passband edges for VBAR filter with $t_{oxide} = 0.35 \cdot t_{LNO}$. **e-f** Power handling tests of a VBAR filter. **e** Filter response measured at low power (-15 dBm) before applying high-power tones in the center of the passband (f_c) and at the band edges (f_l, f_h). **f** Insertion loss at these three frequencies for increasing input power. The filter is destroyed when the input power at the lower band edge (f_l) exceeds 32.6 dBm.

- L. 293 of the revised version:

To complement the TCF and high-power characterization on single resonators (Fig. 3), we characterize the transmission response of the filter for varying temperatures (Fig. 4d). We extract the TCF at the 10 dB edges of the passband. We find that the TCF of the lower edge is comparable to the resonance TCF measured for a single resonator with similar SiO_2 thickness. Furthermore, we assess the power handling capabilities of a VBAR filter by measuring the insertion loss for high-power tones located in the center and at the edges of the passband (Fig. 4e-f). Compared to the previous tests (Fig. 3c-e) where all the input power is directed at a single resonator, the filter topology and frequency of the applied tone heavily

Advanced NEMS Group
EPFL-STI-IGM NEMS
MED 2 2726, Station 9
CH-1015, Lausanne Switzerland

Prof. Guillermo Villanueva
Guillermo.Villanueva@epfl.ch
Ph.: +41 21 693 11 87
http://nems.epfl.ch

influence how much power is dissipated in the individual resonators in the filter[44]. We observe that the IL of the filter gradually starts to change at around 30 dBm of input power, indicating the onset of non-linear behavior. Ultimately, the filter fails completely when the input power applied at the lower passband edge exceeds 32.6 dBm.

Comment 4:

Do you have the data to support the effect of the size of the Si anchor on the quality factor? *need to mention in the manuscript why the measured data were challenging*

Response:

We thank the reviewer for their time in reviewing our paper and giving us suggestions. We did not understand that we had to add this information to the manuscript, but rather that the reviewer wanted clarifications on our work, which we think we provided.

Since we agree that this question may arise for future readers of the work, we have added a sentence that clarifies why the experimental implementation of resonators with different anchor widths was challenging and why we are showing the extreme cases of a directly-mounted and fully released device instead.

Changes:

- L. 220 of the revised version:

  At f_r, the Q is further decreased by the resistive losses of the electrodes, which represent roughly half of the impedance seen at resonance. To experimentally study the effect of different anchor widths on Q (see the simulated trend in Supplementary Fig. S2), we are limited by the etching process, which yields a constant undercut for all resonators on a given chip and is impractical to vary device to device. Instead, we demonstrate the energy confinement mechanism by fabricating the extreme cases where the ridges are either directly mounted to the substrate or fully released (Fig. 2h).

Comment 5:

What is the limitation in improving the selectivity of the filter? *need to show the simulation results to demonstrate the possibility of improving the selectivity*

Response:

We thank the reviewer for their time in reviewing our paper and giving us suggestions. We did not understand that we had to add this information to the manuscript, but rather that the reviewer wanted clarifications on our work, which we think we provided.

The primary objective of this manuscript is to demonstrate the fundamental operation of this novel acoustic resonator and to provide an experimental proof-of-concept for a wide-bandwidth filter. While the current filter design is unoptimized, it successfully serves this demonstrative purpose.

We agree that improving filter selectivity is a critical next step, but we believe that this is out of the scope of this work. To be clear, we are not experts in filter design, so we can only provide rough answers to the reviewers' request. As requested, we have performed simulations to demonstrate how the selectivity of the filter can be improved. Below, we present simulated filter responses assuming different mechanical quality factors of the main mode for the three resonators. These simulations indicate that the width of the transition band and the overall selectivity can be significantly improved if the mechanical Q of the resonators is optimized to > 200 (compared to the Q ≈ 75 of our current fabricated devices).

Advanced NEMS Group
EPFL-STI-IGM NEMS
MED 2 2726, Station 9
CH-1015, Lausanne Switzerland

Prof. Guillermo Villanueva
Guillermo.Villanueva@epfl.ch
Ph.: +41 21 693 11 87
http://nems.epfl.ch

Because the relationship between Q and selectivity in ladder-type acoustic filters is a well-established principle, these simulations align with the standard design expectations. As such, these purely theoretical simulations do not provide anything novel and we feel that including them in the manuscript without accompanying experimental validation would distract from the core, experimental results of this paper. We have provided these simulations here to answer the question but have opted to omit them from the manuscript and maintain the current focus.

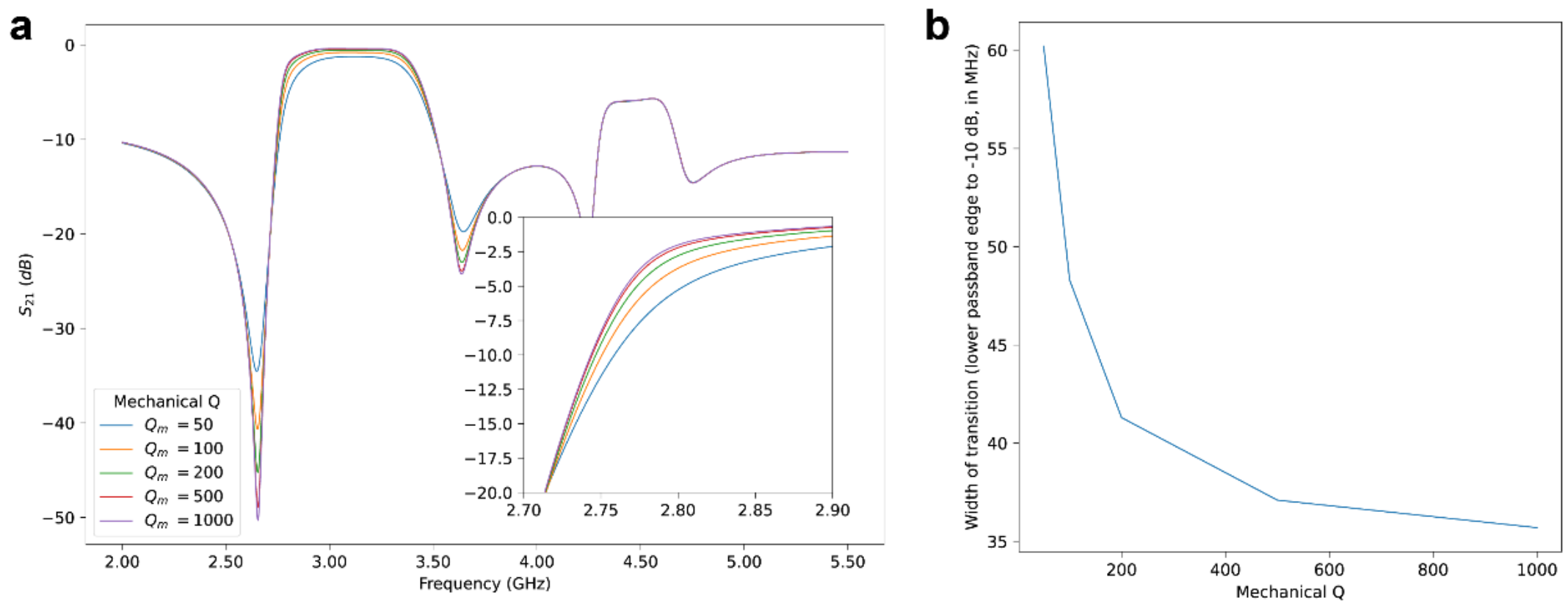

Fig. 1 a Simulated filter responses and enhanced view of the transition region at the lower passband edge. The filter topology corresponds to the filters shown in the manuscript. The resonators are represented by an mBVD circuit model. The motional resistance associated with the main SH-BAW mode is scaled to simulate different mechanical quality factors. **b** Width of the transition band defined as the difference between the passband edge and the frequency where S_{21} reaches -10 dB. A narrower transition indicates a higher selectivity.

Advanced NEMS Group
EPFL-STI-IGM NEMS
MED 2 2726, Station 9
CH-1015, Lausanne Switzerland

Prof. Guillermo Villanueva
Guillermo.Villanueva@epfl.ch
Ph.: +41 21 693 11 87
http://nems.epfl.ch

Manuscript Number: COMMSENG-25-0857B

Title: A bulk acoustic resonator with vertical electrodes for wideband filters

Authors: S. Stettler, E. Navarro-Gesse, C. Collado, J. Mateu, and L. G. Villanueva

June 15th, 2026

Dear Editor and Referees,

Thank you for your continued time and effort in reviewing our manuscript. Based on reviewer feedback, we have not made any additional changes to the content except for modifications according to the checklist provided by the editorial team.

Reviewer #1

The authors have thoroughly addressed the concerns raised in the previous two rounds. The newly added content in the revised manuscript, like the simulated trends for electrode width, the newly measured TCF and high-power data for the filter, all significantly improve the paper. The VBAR concept is novel and promising for wideband 5G/6G filters, and I recommend acceptance for publication

Response:

We thank the reviewer for their thorough evaluation and constructive feedback, which have significantly improved the quality of this manuscript.

Reviewer #3

The authors have addressed all questions. I think the current version can be published

Response:

We sincerely thank the reviewer for their careful reading and comments throughout the review process, which have helped us refine and strengthen the final paper.
