# Supplementary material for: A bulk acoustic resonator with vertical electrodes for wideband filters
Source: Commun Eng. 2026 Jul 21;5:193. doi: 10.1038/s44172-026-00733-1 (PMC13754800; doi:10.1038/s44172-026-00733-1)
Supplement: Supplementary file 2 [file 44172_2026_733_MOESM2_ESM.docx]

Supplementary Information:

A bulk acoustic resonator with vertical electrodes for wideband filters

Silvan Stettler^1^*, Edgar Navarro-Gesse^2^, Carlos Collado^2^, Jordi Mateu^2^, Luis G. Villanueva^1^

^1^ Advanced Nanoelectromechanical Systems Laboratory, École Polytechnique Fédérale de Lausanne (EPFL), 1015 Lausanne, Switzerland

^2^ Signal Theory and Communications Department, Universitat Politècnica de Catalunya (UPC), Castelldefels, Spain

* Corresponding author; [silvan.stettler@epfl.ch](mailto:silvan.stettler@epfl.ch)

## Section 1: Suppression of acoustic energy leakage to the substrate

**Fig. S1 Simplified schematic representation of the resonator geometry.** The annotations show the displacements in the LiNbO_3_ ridge (purple) and the Si pedestal acting as the anchor (light blue/gray). We assume the displacement to be a purely shear horizontal (SH) standing wave.

The general idea to suppress the leakage of acoustic energy is to create an anchor for the ridge in the form of a Si pedestal that does not support the transmission of the SH wave to the substrate. We assume that the resonating mode is a pure SH wave (displacement only along *z*, parallel to the boundaries of the ridge). Further, we simplify the anchor geometry and assume a narrow ridge with width *w_a_* (Fig. S1). In the absence of any mode conversion, the acoustic energy can only leak through the anchor as SH-type waves with a real wave vector component along *z* (*k_z_*). The cut-off frequency of the n-th order SH mode (*f_c_^n^*) in a rectangular waveguide with width *w_a_* is^1^

$$f_{c}^{n}=\frac{n{\cdot v}_{s,Si}}{2\cdot w_{a}}$$

where *v_s,Si_* is the SH bulk wave velocity of Si (around 4600 m/s). Since the node of the displacement of the resonating mode coincides with the center line of the anchor, any transmitted waves in the anchor must be odd (*n* odd). The resonance frequency of the SH-wave in the ridge is approximately

$$f_{r}=\frac{v_{s,LNO}}{2{\cdot w}_{ridge}}$$

where *v_s,LNO_* is the SH bulk wave velocity of LiNbO_3_. To confine the SH wave in the ridge, the resonance frequency must be below the lowest cut-off frequency of the SH wave in the anchor which results in the condition

$$w_{a}<\frac{v_{s,Si}}{v_{s,LNO}}w_{ridge}$$

*v_s,LNO_* is smaller (3490 m/s and 4200 m/s for short and open-circuit boundary conditions, respectively) than *v_s,Si_* . Thus, the resonance frequency is below the cut-off frequency of the anchor even if *w_a_* is equal to *w_ridge_*, and the SH wave in the anchor is evanescent. Provided that the anchor is long enough to avoid evanescent coupling to BAWs in the substrate, the acoustic energy should remain confined in the LiNbO_3_ ridge. It must be noted that this model is only approximative since it completely ignores any displacements associated with in-plane (vertical) shear or longitudinal partial waves.

**Fig. S2 Simulated admittance and eigenmodes for different anchor dimensions.** **a** Simulated admittance for a LiNbO_3_ ridge with three different mounting configurations: mounted directly to the silicon substrate (no anchor), mounted with an anchor with a width *w*_a_ at the narrowest point, or fully released. **b** Eigenmodes corresponding to the admittance peak for different anchor configurations. The displacement is plotted with a logarithmic normalization to emphasize the presence of BAWs in the substrate. **c**-**e** Simulated **c** *Q* at resonance, **d** resonance frequency (*f*_r_), and **e** *k*_t_^2^ as a function of *w*_a_/*w*. For the simulations in this figure, the other dimensions of the resonators are *w*_ridge_ = 500 nm, *w*_e_ = 100 nm, *t*_LNO_ = 400 nm, *t*_oxide_ = 100 nm. *w* denotes the total width of the acoustic cavity (LiNbO_3_ ridge plus twice the electrode width*, w*_ridge_ + 2∙*w*_e_). The intrinsic mechanical *Q* assigned to all materials (imaginary part of stiffness coefficients) is 1000.

In Fig. S2a, we show the simulated admittance of a VBAR unit cell with the ridge mounted directly to the substrate and compared to a configuration with a narrow anchor of various relative widths and a fully released ridge. With the direct mount configuration, the ratio of admittances at resonance and anti-resonance is notably worse than with the narrow anchor configuration. In this case, the energy is only poorly confined, and the resonating ridge acts more like a transducer that radiates acoustic energy into the bulk. The admittance at resonance increases with decreasing anchor width. Simulations of the displacement field (Fig. S2b) show that the displacement amplitude in the bulk, which represents the leakage, indeed decreases the narrower the anchor is. In Fig. S2c, we show the *Q* at resonance as a function of the relative anchor width. For *w*_a_/*w* = 0.1 and the fully suspended ridge, the *Q* approaches the intrinsic *Q* assigned to all materials in the simulation (= 1000). As shown in Fig. S1d-e, the dimensions of the anchor also affect *f*_r_ and *k*_t_^2^. With a narrow anchor, the movement of the LiNbO_3_ is less constrained, which results in higher *k*_t_^2^ and lower *f*_r_.

## Section 2: Dependence of the VBAR response on the electrode width and layer thicknesses

The role of the electrode width (*w*_e_) in a VBAR can be understood in a similar manner as the role of the electrode thickness in a conventional FBAR^2^. Fig. S3a shows the simulated admittance response of a VBAR with varying aluminum electrode width for a fixed ridge width *w*_ridge_ = 500 nm. Widening the electrodes represents an increase in the total width of the acoustic cavity and leads to a reduction in resonance frequency. For *w*_e_ > 0.15∙*w*_ridge_, a significant part of the total elastic energy starts to be concentrated in the electrodes instead of the LiNbO_3,_ which decreases *k*_t_^2^. Based on only the trends of *f*_r_ and *k*_t_^2^ for an Al electrode, *w*_e_ is ideally chosen to be < 0.15∙*w*_ridge_. However, thinner electrodes also increase the resistive losses of the resonator. Considering this trade-off, we target an electrode width of around 75 nm which corresponds to 0.15∙*w*_ridge_ for VBARs with *w*_ridge_ = 500 nm. We note that while *w*_ridge_ can be adjusted lithographically for each device, the absolute electrode width (i.e. 70 nm for the fabricated devices) is controlled by the duration of the Al deposition and thus the same for all devices (independent of *w*_ridge_ ) on a given sample.

**Fig. S3 Simulated dependence of the VBAR response on the width of the Al electrodes (*w*_e_). a** Admittance for varying electrode width. **b** Extracted resonance frequencies and *k*_t_^2^ as a function of *w*_e_. For these simulations, *w*_ridge_ = 500 nm and *t*_LNO_ = 400 nm.

Fig. S4a shows the simulated admittance response of a VBAR with varying LiNbO_3_ thickness (*t*_LNO_). Fig. S4b shows the extracted resonance frequencies of the fundamental mode and first thickness harmonic spurious mode. *t*_LNO_ strongly influences the frequencies of the spurious thickness harmonics that appear after the fundamental resonance frequency. For LiNbO_3_ films thinner than 1 µm, the presence of the Si anchor has a stiffening effect (higher *f*_r_) on the structure. Within that range, *t*_LNO_ has a non-negligible impact on the acoustic phase velocity and thus *f*_r_. As *t*_LNO_ becomes larger (*t*_LNO_ > 1 µm), *f*_r_ becomes nearly invariant to changes in *t*_LNO_ and is mainly dependent on the width and acoustic properties of the LiNbO_3_ and electrodes. Fig. S4c-d analyzes the impact of adding a SiO_2_ layer on top of the LiNbO_3_ ridge with a constant thickness. Like increasing *t*_LNO_, adding SiO_2_ also brings the first thickness harmonic spurious mode closer to the main resonance.

Furthermore, the thicknesses of the constituent layers of the ridge have a strong impact on the static capacitance (*C*_0_) of the resonator. We approximate a single ridge composed of only LiNbO_3_ (with permittivity ε_LNO_) as a parallel-plate capacitor with an electrode area given by *t*_LNO_∙*L*, where *L* is the aperture or ridge length. *C*_0_ of a VBAR composed of *N* parallel-connected ridges is estimated as

$$C_{0}\approx\epsilon_{LNO}\frac{{N\cdot t}_{LNO}\cdot L}{w_{ridge}}$$

The design of ladder filters typically requires specific *C*_0_ values to achieve good impedance matching and rejection. For conventional BAW and SAW resonators, this constrains resonator and filter size as *C*_0_ is proportional to the planar dimensions of the resonator. For VBARs on the other hand, *C*_0_ can in principle be scaled using the third dimension by changing *t*_LNO_.

**Fig. S4 Simulations showing the dependence of the admittance response on the layer thicknesses.** **a** Admittance for varying LiNbO_3_ thicknesses (without a SiO_2_) layer. As *t*_LNO_ increases, spurious thickness harmonics move closer to the fundamental resonance. **b** Resonance frequencies of the fundamental and the first thickness harmonic. **c** Admittance for a fixed *t*_LNO_ = 400 nm and varying SiO_2_ thickness (*t*_oxide_). In contrast to the case without SiO_2_ shown in **a-b**, the first thickness harmonic (ripple above 4 GHz) is stronger. **d** Resonance frequencies of the fundamental and first thickness harmonic for varying *t*_oxide_. For these simulations, *w*_ridge_ = 500 nm and *w*_e_ = 100 nm.

## Section 3: Additional resonator measurements

**Fig. S5 Measurements of VBARs with varying aperture lengths.** The measurements are shifted on the vertical axis for clarity. The spacing between the ripples appearing between *f*_r_ and *f*_ar_ becomes larger for shorter apertures, which confirms that those ripples are transversal modes.

## Section 4: VBAR fabrication

Fig. S6 shows a schematic outline of the process used to fabricate the resonators and filters. For the initial development of the individual steps, we used dummy Si samples, which we cleave to reveal the cross-sectional geometry. Fig. S7 shows the main steps on dummy samples without a LiNbO_3_ film. For the IBE step (Fig. S6b), we use a LiNbO_3_-on-Si test chip to characterize and validate the IBE process that patterns the ridges (Fig. S8). Fig. S9 shows the result of the optional RIE process we use to remove the SiO_2_.

**Fig. S6 Schematic outline of the process flow used to fabricate the resonators and filters. a** SiO_2_ deposition and patterning using e-beam lithography and ICP-based dry etching (CHF_3_/H_2_/He chemistry). We use e-beam lithography to ensure high uniformity of the ridge width (*w*_ridge_). **b** IBE with a 45° ion incidence angle for vertical sidewalls to define the LiNbO_3_ ridges. **c** Optical lithography and lift-off for the Al pads. **d** Al sputtering followed by **e** IBE with near-vertical ion incidence (10°) to remove the Al only on horizontal surfaces. **f** Partial undercut of the ridges with dry etching (ICP SF_6_ with a low frequency wafer voltage bias) to define the Si pedestals. The spacing between adjacent ridges (*s*) is the same everywhere to improve the uniformity of this process and minimize variations in the width of the Si pedestal.

**Fig. S7 Process development on dummy samples. a** Development of the main process steps to fabricate the ridges on a dummy Si sample without a LiNbO_3_ layer. **b** Test sample (patterned SiO_2_ ridges on Si wafer, no sidewall electrodes) to showcase the dry etching process (ICP SF_6_ with a low frequency wafer voltage bias) for the partial release of the ridges. The width of the Si pedestal under the ridge can be controlled with etch duration. The recipe is designed for deep anisotropic etching of silicon but features a consistent undercut that correlates with the duration of the etch pulse. If the pulse is too long (7 sec.), the undercut is too large, and the ridge is fully released. The etch rate of this process also depends on the exposed area between two ridges. Thus, the spacing between ridges is kept the same for all fabricated devices to maximize the uniformity of the etch rate.

**Fig. S8 Details on the LiNbO_3_ etching processes. a** Cleaved test sample after IBE of a thin LiNbO_3_ film with the wafer fixture set for an ion incidence angle of 45° and a SiO_2_ hard mask. With this fixture angle, the etch rate is significantly faster in the center of large features, but as like with the initial Si dummy sample (Fig. S7), the sidewalls are vertical. **b** Tilted side view of the etched ridge showing the sidewalls of the SiO_2_ hard mask and the underlying LiNbO_3_.

**Fig. S9 Ridge geometry before and after removing the SiO_2_ layer.** SEM images and approximate cross-sectional geometries of the ridge **a** with the SiO_2_ layer on top of the LiNbO_3_ and **b** after removing the SiO_2_ layer. The electrodes protrude above the LiNbO_3_ top surface (instead of being flush) because they are not etched significantly by the dry etching process (RIE with C_4_F_8_/H_2_/He chemistry) used to remove the SiO_2_. Scale bar 500 nm.

## Section 5: Power handling and IMD3 measurements

Fig. S10a shows a block diagram of the 3-port setup used for power handling measurements. The input tone at *f*_0_ from the signal generator (SG) passes through a high power amplifier and is transmitted to port 2 where a GSG probe and the device under test (DUT) are connected. The two circulators between the amplifier and the GSG probe isolate the amplifier output from the reflections of the DUT. The reflected signal at the DUT is transmitted towards a last circulator to attenuate and protect the spectrum analyzer (SA). Prior to measurements, we measure 3-port S-parameters (with the ports as defined on Fig. S10a) of the setup with a vector network analyzer (VNA). We assume that the SG and SA ports are perfectly matched. Using a signal flow graph, we relate the input power set with the signal generator (*P*_SG_) to the outgoing power measured with the spectrum analyzer (*P*_SA_). The power received by the SA (port 3) depends on the S-parameters of the setup and the reflection coefficient Γ at port 2 (GSG probe plus the DUT):

*P*_SA_ = |*S*_31_ + *S*_21_*S*_32_Γ/(1-*S*_22_ Γ)|^2^·*P*_SG_

We solve this equation numerically to find Γ. Γ represents the reflection coefficient of the DUT (= *S*_11_) plus a small influence of the GSG probe, though the probe’s good match and low insertion loss make this effect minimal. The incident power to the GSG probe is calculated as *P*_in_ = |*S*_21_|^2^*P*_SG_.

For transmission measurements on a filter (Fig. S10b), we use a second GSG probe to connect port 2 of the filter to a 20 dB attenuator and the SA. We calculate the filter insertion loss by comparing *P*_SA_ to the *P*_SA_ values measured with a thru structure in the place of the filter. The incident power to the filter is estimated as *P*_in_ = |*S*_21_|^2^*P*_SG_.

Fig. S10c shows a block diagram of the 4-port setup used for the IMD3 measurements on single resonators. Prior to measurements, we measure 4-port S-parameters (with the ports as defined on Fig. S10c) of the setup with a VNA. The two input tones *f*_1_ and *f*_2_ (with power *P*_SG_ each) are amplified and pass through a low-pass filter to remove the harmonics generated in the amplifiers. The two tones are combined with a Wilkinson combiner and transmitted towards the GSG probe and DUT with a hybrid coupler. The hybrid coupler directs the reflected power from port 3 to the SA. The incident power to the GSG probe (port 3) is *P*_in_ = |*S*_31_|^2^*P*_SG._ The power of the reflected fundamental and IMD3 tones at port 3 is calculated as *P*_refl_(*f*) = *P*_SA_(*f*) / |*S*_43_|^2^.

**Fig. S10 Experimental setups for high power and IMD3 measurements. a** 3-port setup with one input tone *f*_0_ used to measure the reflection coefficient Γ of a one-port DUT under high power. **b** For filters, we measure transmission by connecting port 2 of the filter to the SA **c** 4-port setup with two input tones *f*_1_ and *f*_2_ used to measure IMD3 in the reflected signal.

## References

1.	Auld, B. A. *Acoustic Fields and Waves in Solids: Volume II*. (John Wiley & Sons, 1973).

2.	Hashimoto, K. *RF Bulk Acoustic Wave Filters for Communications*. (Artech House, 2009).
